# Super-resolution mapping of the ankle link proteins ADGRV1 and PDZD7 in developing auditory hair cells

**DOI:** 10.1016/j.isci.2025.113190

**Published:** 2025-07-24

**Authors:** Baptiste Colcombet-Cazenave, Gaël Moneron, Antonio El Helou, David DiGregorio, Vincent Michel, Nicolas Wolff

**Affiliations:** 1 Institut Pasteur, Université Paris Cité, CNRS UMR 3571, Signaling and Dynamic of Receptors Unit, F-75015 Paris, France; 2 Sorbonne Université, F-75005 Paris, France; 3 Department of Physiology and Biophysics, University of Colorado School of Medicine, Aurora, CO 80045, USA; 4 Institut Pasteur, Université Paris Cité, INSERM, Pathogenesis of Vascular Infections Unit, F-75015 Paris, France

**Keywords:** Molecular neuroscience, Cellular neuroscience, Sensory neuroscience

## Abstract

Auditory hair cells convert mechanical stimuli into electrical signals. Inner hair cells (IHCs) serve as primary sensory receptors, while outer hair cells (OHCs) function as cochlear amplifiers. During development, the G-protein-coupled receptor ADGRV1, along with usherin, whirlin, and PDZD7, interacts at the base of stereocilia to form the transient ankle-link complex. The long extracellular domain of ADGRV1 bridges adjacent stereocilia, while its intracellular domain binds to whirlin and PDZD7. Using STED nanoscopy on juvenile mouse hair cells, we mapped the spatial distribution of ADGRV1 regions and PDZD7, revealing highly asymmetric localization patterns within stereocilia rows and between IHCs and OHCs. These distinct distributions reflect tightly regulated subcellular targeting of the proteins, which show strong colocalization. Interestingly, while the extracellular portion of ADGRV1 is no longer detectable after postnatal day 12, the GPCR domain persists until P21, suggesting that ADGRV1 may also play a signaling role beyond its scaffolding function.

## Highlights

• Mapping ankle link proteins by averaging STED images of hair bundle stereocilia
• PDZD7 exclusively colocalizes with ADGRV1’s cytoplasmic domain in stereocilia
• In OHCs, ADGRV1 spans inter-row spaces between stereocilia
• In IHCs, ADGRV1 mainly localizes to the tallest row of stereocilia

## Graphical abstract

**Figure:**
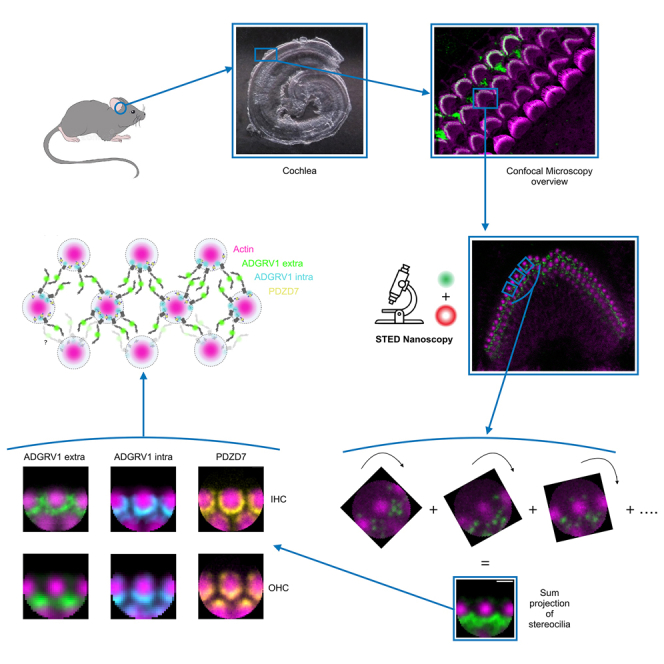

## Introduction

Cochlear hair cells are the primary sensors for sound detection in mammalian inner ear. They possess at their apex a bundle of actin-based stereocilia organized in rows of increasing height that are deflected by sound-induced vibrations. Along the axis of the cochlea, sound-evoked mechanical stimuli are converted into electrical signals in the hair bundles of two types of sensory hair cells: the inner hair cells (IHCs), which are the genuine sensory cells that stimulate the auditory nerve, and the outer hair cells (OHCs), which serve as cochlear amplifiers. Within each hair cell, the hair bundle is composed of three rows of actin-based microvilli, called the stereocilia, whose spatial organization and mechanicals properties play a critical role in the mechanoelectrical transduction (MET) process.

During early postnatal stages, a complex of four proteins is transiently expressed at the base of the hair cell stereocilia and has been identified as necessary for the normal morphogenesis of the hair bundle.^1^^,^^2^ This complex includes the two transmembrane proteins usherin and ADGRV1 (coded by the genes *USH2A* and *USH2C*, respectively), thought to be the main components of the ankle link that tether neighboring stereocilia, as well as the two cytoplasmic proteins whirlin and PDZD7 (coded by the genes *USH2D* and *PDZD7*, respectively). Variants of these four proteins are associated with non-syndromic as well as syndromic forms of hearing loss, such as the Usher 2 syndrome, which is the most common form of hereditary hearing-vision loss in humans.

The function of usherin, a single-pass transmembrane protein with a large extracellular region (5,011 residues) and a cytoplasmic domain (139 residues) exhibiting a C-terminal PDZ (Postsynaptic density 95, PSD-95; Discs large, Dlg; Zonula occludens-1, ZO-1) binding motif (PBM) remains mostly unknown. ADGRV1 is a seven-pass transmembrane protein of the adhesion G-protein-coupled receptor (aGPCR) family B2.^3^^,^^4^ It is composed of a large extracellular region (5,891 residues), a transmembrane GPCR region, and a cytoplasmic domain (152 residues) with a C-terminal PBM. The long extracellular regions of usherin and ADGRV1 are thought to form transient basal links bridging adjacent stereocilia, referred to as ankle links. These fibrous ankle links have been observed transiently at the base of stereocilia during the postnatal days (∼P2 to ∼ P12) of hair bundle development in mice using transmission electron microscopy but also confocal microscopy with antibody directed against the N-terminal, extracellular region of ADGRV1.^5^^,^^6^^,^^7^ However, the individual contributions of usherin and ADGRV1 to the fibrous ankle links remained unclear. In addition, ADGRV1 possesses an autoproteolysis-inducing domain (GAIN) upstream of its GPCR transmembrane region.^8^ The GAIN domain of adhesion GPCRs can catalyze the autoproteolysis of the protein, allowing to separate its extracellular region (α subunit; ADGRV1α) from the transmembrane and cytoplasmic domains (β subunit; ADGRV1β) under environmental constraints. The new N-terminal end of the β subunit may act as a tethered agonist peptide and activate the GPCR to trigger and/or switch cellular signaling.^9^ Therefore, ADGRV1 can have a dual role in the morphogenesis and the signaling of hair cells according to the association state of its α and β subunits.

At the anchoring sites of the ankle links, the cytoplasmic C-terminal PBMs of usherin and ADGRV1 associate to the two paralogous proteins whirlin and PDZD7 by binding to their N-terminal PDZ domains.^10^ While whirlin is widely expressed at the apex of the hair cell stereocilia, it is also transiently detected, conjointly with PDZD7, at the ankle-links level.^11^ These two cytoplasmic proteins are thought to promote the assembly of a large cytoplasmic network through protein-protein interactions mainly mediated by PDZ domains and harmonin homology domains (HHDs). Moreover, their C-terminal regions associate to actin-binding proteins, thus strongly anchoring the system to actin filaments in the center of the stereocilia.^12^^,^^13^ This network of interactions determines the distribution of the ankle link proteins at the basis of stereocilia. Knockout of PDZD7 in mouse results in a loss of whirlin labeling at the ankle links and a diffused localization of ADGRV1 and usherin, suggesting that it acts as a primary scaffold for the Usher 2 complex assembly.^14^ PDZD7 has also been shown as suppressing ADGRV1β signaling in cellular models.^9^

If the spatial distribution of the ankle link complex components has been described as being localized at the base of the hair bundles, it has poorly been characterized so far in terms of precise localization between and within rows of stereocilia due to the respective limitations of the previously used imaging techniques. Furthermore, the fine spatial localization differences of distinct proteins across stereocilia rows in both OHCs and IHCs remain unexplored, as does the potential dissociation of ADGRV1 α and β subunits, despite being of significant relevance for hair cell development and signaling. Indeed, conventional light microscopy suffers from a diffraction limited resolution (∼200 nm) that prohibits a fine mapping of any inter-stereocilia macromolecular organization, and electron microscopy suffers from the tediousness of sample preparation, a low throughput for sampling a large number of cells and stages of hair cell development and the potential difficulty of labeling unambiguously multiple molecules of interest. To circumvent those limitations, we have used state-of-the-art stimulated emission depletion (STED) fluorescence microscopy, a unique fluorescence imaging technique providing tens of nanometers of spatial resolution, simultaneously for multiple channels with molecular specificity, in whole organ of Corti preparations.^15^^,^^16^ To reveal the nanoscale distribution of ADGVR1 and its regulator PDZD7, we have developed a custom image analysis workflow based on image averaging of single stereocilia. This mapping was necessary to establish a model of ankle links distribution in the hair bundles of mouse cochlear sensory cells, providing insights into spatial structure of ankle links and their influence on hair bundle morphogenesis during the development. In addition, we have monitored the localization of the ADGRV1 α and β subunits over time, revealing the persistence of ADGRV1 β subunit after disappearance of ADGRV1 α together with the ankle links after P12, thus potentially inducing a timed switch in cellular signaling induced by the GPRC moiety of the protein.

## Results

### Mapping of ADGRV1 and PDZD7 proteins using super resolution microscopy

In order to precisely identify patterns of organization of ADGRV1 and PDZD7 at different stages of development, we employed state-of-the-art stimulated emission depletion (STED) super-resolution fluorescence microscopy. While conventional confocal microscopy approaches typically produce images with a resolution around 200 nm in the XY plan, we performed multicolor super-resolution imaging of probes targeting ankle-links-associated proteins at 40 to 60 nm planar resolution on hair bundles of whole mouse organ of Corti explants (conservative resolution estimated from the width of line profiles of the smallest structures found in the images). We used polyclonal antibodies directed against the extracellular region of ADGRV1 (ADGRV1extra), the cytoplasmic domain of ADGRV1 (ADGRV1intra), as well as against the cytoplasmic protein PDZD7. ADGRV1-extra antibodies target epitopes in positions 2669–2812 in human ADGRV1 (UniProtKB: Q8WXG9), meaning in the middle of the extracellular region α subunit that is a component of the ankle links. ADGRV1-intra antibody recognizes the cytoplasmic domain of β subunit. The two antibodies targeting ADGRV1 were validated by comparing the immunolabeling of the protein in wild-type mice with the immunolabeling in *Adgrv1*^tm1Msat^(*adgrv1*^−/−^) knockout mice, thanks to Dr. H. Sato (University of Osaka, Japan).^17^^,^^18^ We clearly observed the absence of ADGRV1 staining, using our antibodies targeting the extra- (Figure S1) and intra-cellular (Figure S2) regions of the protein, in the knockout mice *adgrv1*^−/−^ compared to wild-type mice for both IHCs and OHCs. Since we did not have access to PDZD7 knockout mice, we validated the anti-PDZD7 antibody in HEK cells. These cells were transfected under the same experimental conditions, either with a pMT3 plasmid expressing a GFP-PDZD7 fusion protein or with a pMT3 plasmid expressing only the protein GFP. The transfected cells were then labeled with the primary antibody. Images of confocal microscopy were collected using the same acquisition parameters, and no labeling was observed in cells transfected with the GFP-only plasmid, while cells transfected with the GFP-PDZD7 fusion plasmid showed clear labeling (Figure S3). We concluded that the antibody provided by *MyBioSource* (#MBS3217600) is specific for PDZD7 in this cellular experiment.

**Figure 1:**
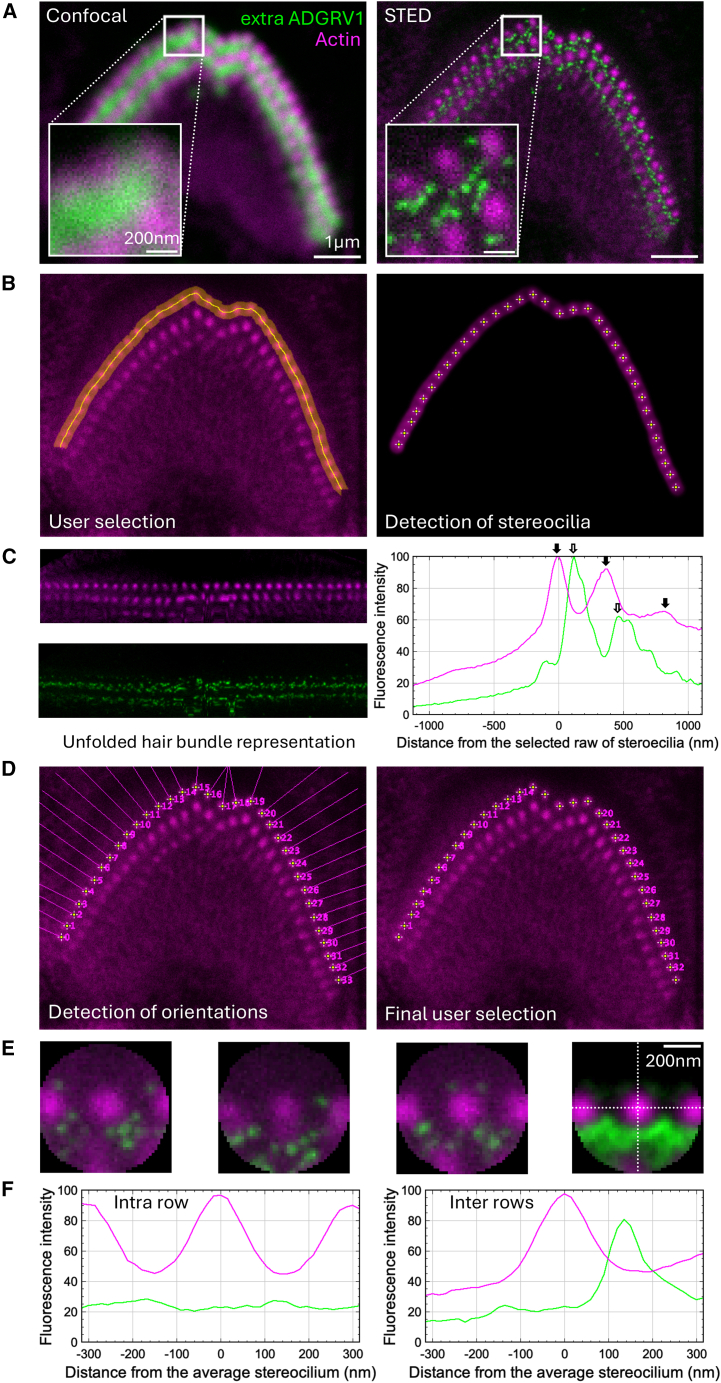
Analysis procedure of STED images (A) Images of confocal microscopy (left) and STED microscopy (right) recorded on the same microscope, on the same OHC hair cell at P5. Scale bars, 1 μm (200 nm in the enlarged views). (B) Manual masking of the cell’s outer stereocilia row (left) and automatic detection of the center of each stereocilia (right). (C) Unfolded bundles (left) with actin labeled in purple and ADGRV1 in green. Distribution graph of the normalized intensity profiles of fluorescence for the two colors. The origin of the *x* axis (distance 0 nm) corresponds to the outer row. Arrows indicate the maxima of fluorescence. (D) Representation of the automated calculation of stereocilia orientations (left) and user selection of stereocilia to sum (right). (E) Three individual stereocilia extracted after reorientation and sum of aligned stereocilia selected in (D) (right). (F) Fluorescence intensity profiles of actin and ADGRV1 are plotted along the horizontal stereocilia axis (left) and along the vertical stereocilia axis (right), illustrating the intra- and inter-row distributions, respectively.

The gain in resolution from confocal to STED microscopy in our experimental conditions is illustrated in Figure 1A on the outer hair cell at postnatal P5. In confocal microscopy (left image), labeling of ADGRV1 extracellular region (in green) appears to be homogeneously distributed between the rows of stereocilia (in magenta). In STED microscopy (right image), on the same cell recorded simultaneously (line interleaving, see image acquisition section), discrete spots are revealed and allow for an accurate and precise inter-stereocilia localization of the probe. To extract characteristic patterns of spatial distributions of our proteins around stereocilia and account for the curvature of hair bundles, we have developed a semi-automated approach to analyze STED images, as illustrated with the same outer hair cell in Figures 1B–1F. The workflow is described in detail in the image analysis section. First, manual masking of the cell’s outer stereocilia row (Figure 1B, left) allows to automatically find the center of each stereocilia (Figure 1B, right). Coordinates of these stereocilia are used to unfold the whole hair bundle (Figure 1C, left). Average transverse profiles across the whole bundle, orthogonal to the row of cilia, are obtained by horizontal projections of the two channels respectively (Figure 1C, right). The origin of the x axis (distance 0 nm) systematically corresponds to the outer row in all graphs of this study. This representation of the relative distribution of the protein of interest around the cilia gives an indication of the presence of inter- and intra-row links between cilia, as well as the proportions of their occurrences. One has to keep in mind that the distortion of space used to straighten the hair bundle induces inaccuracy in distances in the unfolded images, along any lines parallel to the row of cilia due to expansion or compression of space (reason for the absence of a scale bar on the straighten representations). However, the distortion of space does not significantly affect distances in the transverse profiles that are averaged. Here (Figure 1C), image analysis highlights three local maxima in magenta, corresponding to actin labeling of the three stereocilia rows of the OHC (solid arrows), as well as two main maxima in green, corresponding to inter-row labeling of ADGRV1 extracellular region (arrows). Our workflow requires single stereocilia averaging to reflect protein distributions between all closest neighboring cilia. To this end, we automatically calculated stereocilia orientations with respect to the hair bundle’s curvature, for later angular alignment (Figure 1D, left), and manually discarded non-representative cilia from the averaging (Figure 1D, right), as for example, the stereocilia of the kinked region in the center of the bundle, which display peculiar protein distributions compared to the rest of the bundle and should be described separately as the spatial organization of their closest neighbors (stereocilia), is very much different from all others, which would compromise the final representation of the average stereocilia. These few central stereocilia are in close proximity to the kinocilium during hair bundle maturation, which most likely affects the network of surface proteins. Single stereocilia can be extracted from the hair bundle image with a normalized orientation (normal to the curvature of the bundle), as illustrated by the first three individual stereocilia displayed in Figure 1E. Then, by overlapping and averaging single stereocilia (Figure 1E, right), it is possible to visualize the overall ADGRV1 distribution around the actin core with a better signal-to-noise ratio. Finally, fluorescence intensity profiles along the *x* (horizontal, Figure 1F, left) and *y* (vertical, Figure 1F, right) stereocilia axes are plotted, making for an easy intra- and inter-row distribution analysis (Figure 1F, left and right, respectively).

The images shown in Figures 1, 2, 3, 4, 5, 6, 7, 8, 9 and 10 are representative of what we observed in the numerous cochleae analyzed. For each labeling (PDZD7, extra- and intra-cellular ADGRV1 regions), STED images of additional hair bundles are presented in supplementary data to assess the distribution of the observed proteins in different IHC and OHC cells, different cochleae, and different mice at P5 (Figure S4).

**Figure 2:**
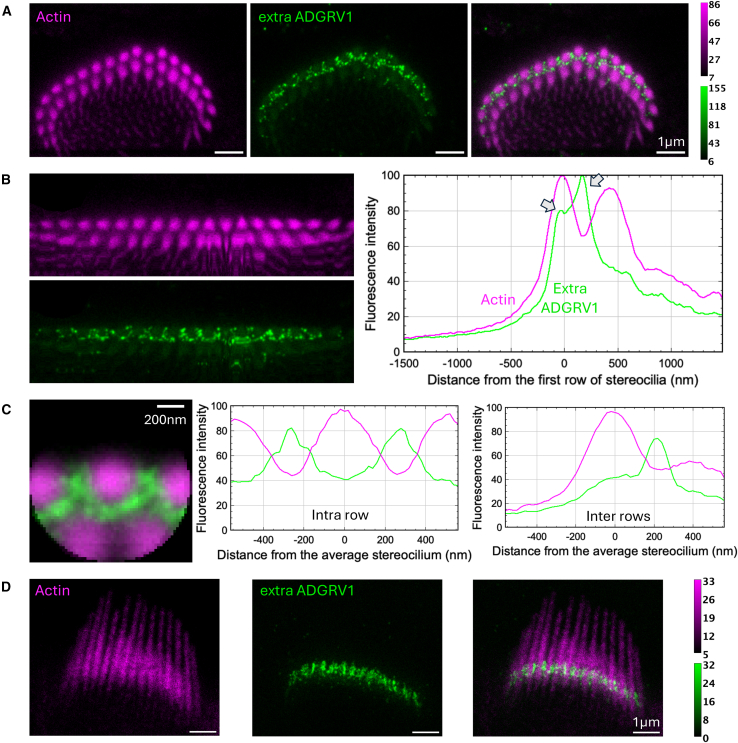
Immunolabeling of ADGRV1 extracellular region in P5 mouse IHCs Two-color STED microscopy imaging of actin colored in purple and ADGRV1 in green. The intensity scales are reported for the two colors. (A) STED images of one IHC with labeling of actin (left), ADGRV1 (center), and composite image (right). Top views. Scale bars, 1 μm. (B) Unfolded images of the IHC hair bundle with labeling of actin (left upper) and ADGRV1 (left lower). The distribution graph of each labeling obtained by projection of the unfolded images (right). Arrows indicate local maxima of fluorescence for ADGRV1. (C) Sum projection of aligned individual stereocilia after normalization of their orientation (left); scale bars, 200 nm. Fluorescence intensity profiles of actin and ADGRV1 are plotted along the horizontal stereocilia axis (center) and along the vertical stereocilia axis (right), illustrating the intra- and inter-row distributions, respectively. (D) STED images of one IHC with labeling of actin (left), ADGRV1 (center), and composite image (right). Lateral views. Scale bars, 1 μm.

**Figure 3:**
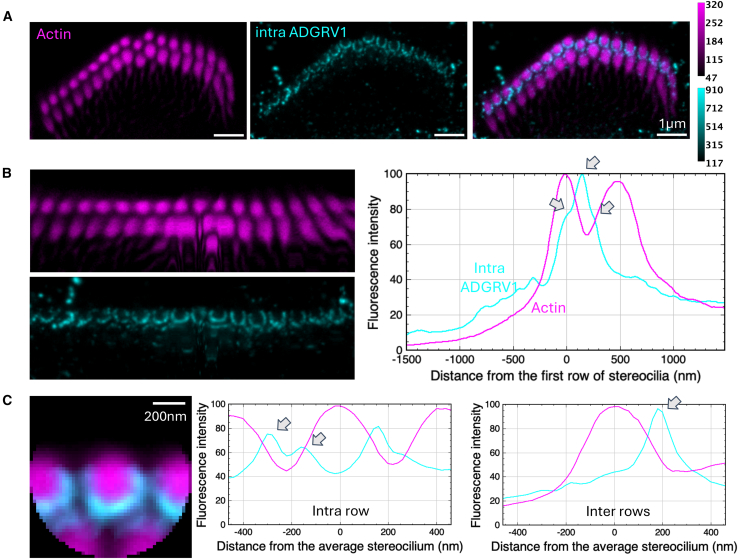
Immunolabeling of ADGRV1 intracellular region in P5 mouse IHCs Two-color STED microscopy imaging of actin colored in purple and ADGRV1 in cyan. Top views. (A) STED images of one IHC with labeling of actin (left), ADGRV1 (center), and composite image (right). Scale bars, 1 μm. The intensity scales are reported for the two colors. (B) Unfolded images of the IHC hair bundle with labeling of actin (left upper) and ADGRV1 (left lower). The distribution graph of each labeling obtained by projection of the unfolded images (right). Arrows indicate local maxima of fluorescence for ADGRV1. (C) Sum projection of aligned individual stereocilia after normalization of their orientation (left); scale bars, 200 nm. Fluorescence intensity profiles of actin and ADGRV1 are plotted along the horizontal stereocilia axis (center) and the vertical stereocilia axis (right), illustrating the intra- and inter-row distributions of proteins, respectively. Arrows indicate local maxima of inter-row fluorescence for ADGRV1.

**Figure 4:**
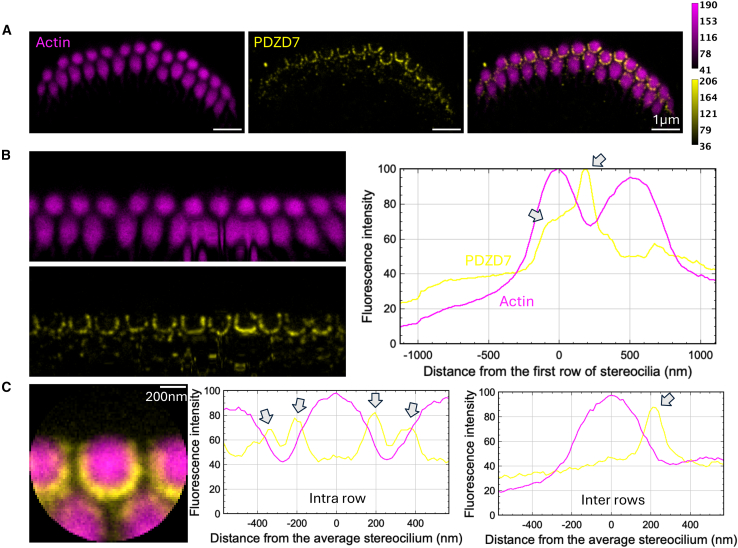
Immunolabeling of PDZD7 in P5 mouse IHCs Two-color STED microscopy imaging of actin colored in purple and PDZD7 in yellow. Top views. (A) STED images of one IHC with labeling of actin (left), PDZD7 (center), and composite image (right). Scale bars, 1 μm. The intensity scales are reported for the two colors. (B) Unfolded images of the IHC hair bundle with labeling of actin (left upper) and PDZD7 (left lower). The distribution graph of each labeling obtained by projection of the unfolded images (right). Arrows indicate local maxima of fluorescence for PDZD7. (C) Sum projection of individual stereocilia after normalization of their orientation (left); scale bars, 200 nm. Fluorescence intensity profiles of actin and PDZD7 are plotted along the horizontal stereocilia axis (center) and the vertical stereocilia axis (right), illustrating the intra- and inter-row distributions of proteins, respectively. Arrows indicate local maxima of fluorescence for PDZD7.

**Figure 5:**
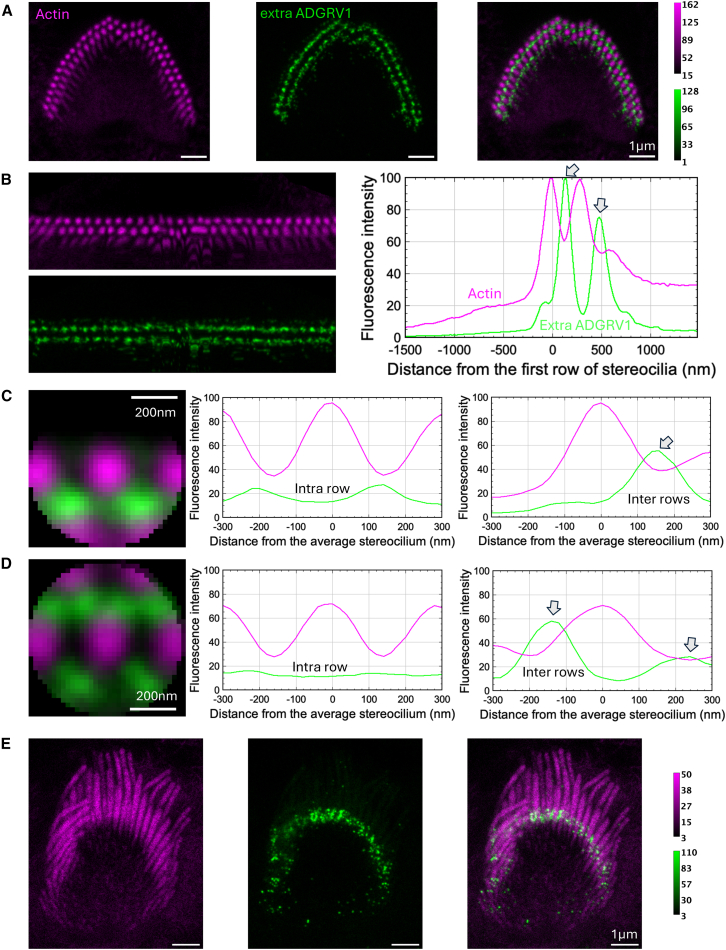
Immunolabeling of ADGRV1 extracellular region and actin in P5 mouse OHCs Two-color STED microscopy imaging of actin colored in purple and ADGRV1 in green. The intensity scales are reported for the two colors. (A) STED images of one OHC with labeling of actin (left), ADGRV1 (center), and composite image (right). Top views. Scale bars, 1 μm. (B) Unfolded images of the OHC hair bundle with labeling of actin (left upper) and ADGRV1 (left lower). The distribution graph of each labeling obtained by projection of the unfolded images (right). Arrows indicate local maxima of fluorescence for ADGRV1. (C) Sum projection of aligned individual stereocilia of the 1^st^ row after normalization of their orientation (left); scale bars, 200 nm. Fluorescence intensity profiles of actin and ADGRV1 are plotted along the horizontal stereocilia axis (center) and the vertical stereocilia axis (right), illustrating the intra- and inter-row distributions of proteins, respectively. The arrow indicates local maximum of fluorescence for ADGRV1. (D) Sum projection of aligned individual stereocilia of the 2^nd^ row after normalization of their orientation (left); scale bars, 200 nm. Fluorescence intensity profiles of actin and ADGRV1 are plotted along the horizontal stereocilia axis (center) and the vertical stereocilia axis (right), illustrating the intra- and inter-row distributions of proteins, respectively. The arrow indicates local maximum of fluorescence for ADGRV1. (E) STED images of one OHC with labeling of actin (left), ADGRV1 (center), and composite image (right). Lateral views. Scale bars, 1 μm.

**Figure 6:**
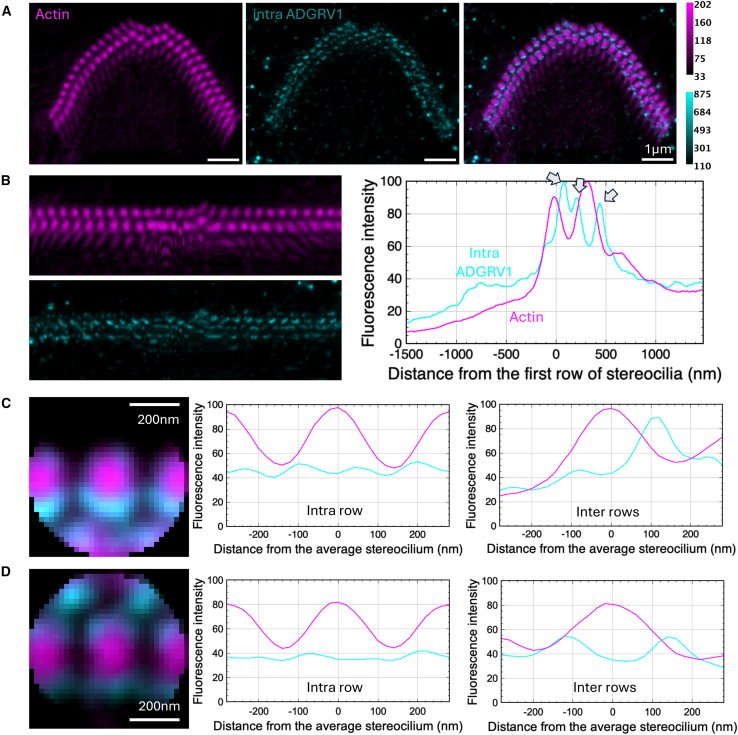
Immunolabeling of ADGRV1 intracellular region in P5 mouse OHCs Two-color STED microscopy imaging of actin colored in purple and ADGRV1 in cyan. Top views. (A) STED images of one OHC with labeling of actin (left), ADGRV1 (center), and composite image (right). Scale bars, 1 μm. The intensity scales are reported for the two colors. (B) Unfolded images of the OHC hair bundle with labeling of actin (left upper) and ADGRV1 (left lower). The distribution graph of each labeling obtained by projection of the unfolded images (right). Arrows indicate local maxima of fluorescence for ADGRV1. (C) Sum projection of aligned individual stereocilia of the 1^st^ row after normalization of their orientation (left); scale bars, 200 nm. Fluorescence intensity profiles of actin and ADGRV1 are plotted along the horizontal stereocilia axis (center) and the vertical stereocilia axis (right), illustrating the intra- and inter-row distributions of proteins, respectively. (D) Sum projection of aligned individual stereocilia of the 2^nd^ row after normalization of their orientation (left); scale bars, 200 m. Fluorescence intensity profiles of actin and ADGRV1 are plotted along the horizontal stereocilia axis (center) and the vertical stereocilia axis (right), illustrating the intra- and inter-row distributions of proteins, respectively.

**Figure 7:**
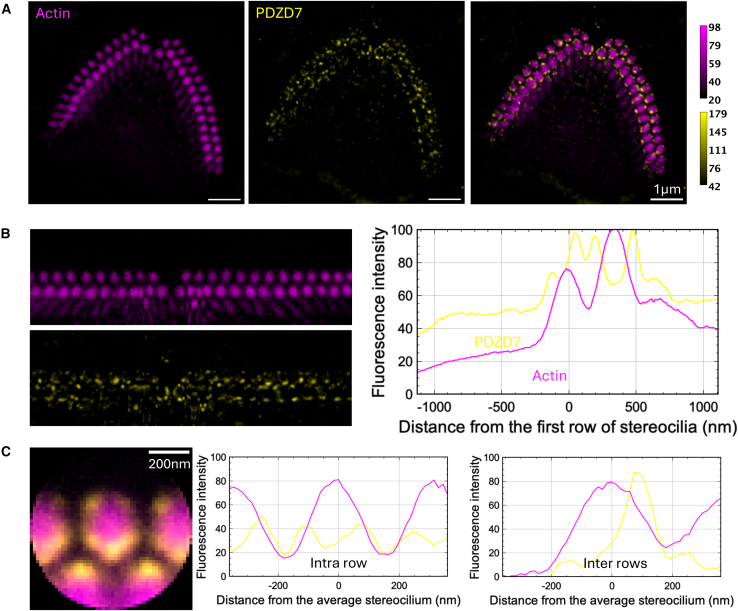
Immunolabeling of PDZD7 in P5 mouse OHCs Two-color STED microscopy imaging of actin colored in purple and PDZD7 in yellow. Top views. (A) STED images of one OHC with labeling of actin (left), PDZD7 (center), and composite image (right). Scale bars, 1 μm. The intensity scales are reported for the two colors. (B) Unfolded images of the OHC hair bundle with labeling of actin (left upper) and PDZD7 (left lower). The distribution graph of each labeling obtained by projection of the unfolded images (right). (C) Sum projection of aligned individual stereocilia after normalization of their orientation (left); scale bars, 200 nm. Fluorescence intensity profiles of actin and PDZD7 are plotted along the horizontal stereocilia axis (center) and the vertical stereocilia axis (right), illustrating the intra- and inter-row distributions of proteins, respectively.

**Figure 8:**
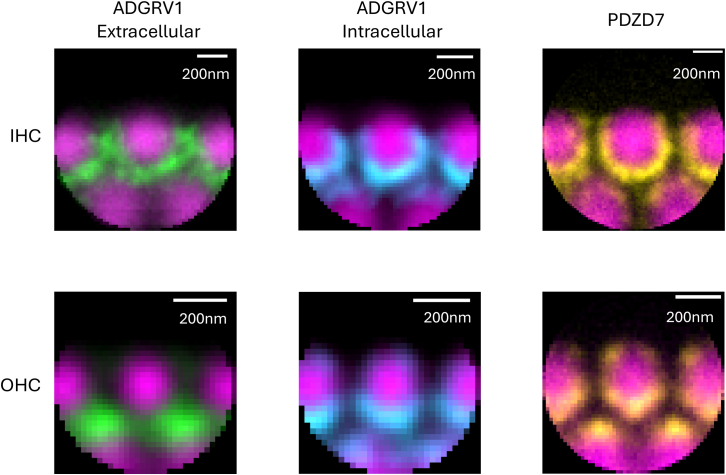
Sum projection of aligned individual stereocilia of the 1^st^ row in P5 mouse IHCs (upper row) and OHCs (lower row) of actin and ADGRV1 extracellular (left column), actin and ADGRV1 intracellular (center column), and actin and PDZD7 (right column). Scale bars, 200 nm.

**Figure 9:**
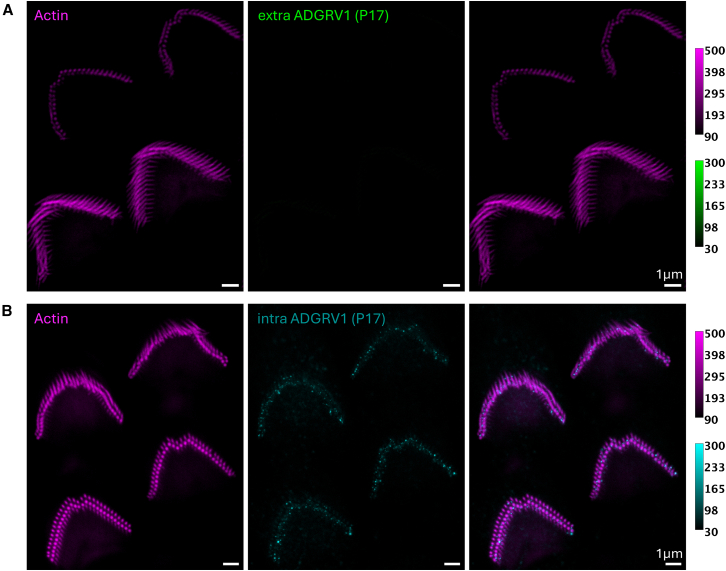
Immunolabeling of ADGRV1 extracellular and intracellular regions in P17 mouse OHCs monitored by two-color STED microscopy Scale bars, 1 μm. The intensity scales are reported for the two colors. (A) Top views of four P17 OHCs STED images with labeling of actin (left, purple), ADGRV1 extracellular region (center, green), and composite image (right). (B) Top views of four P17 OHCs STED images with labeling of actin (left, purple), ADGRV1 intracellular region (center, cyan), and composite image (right).

**Figure 10:**
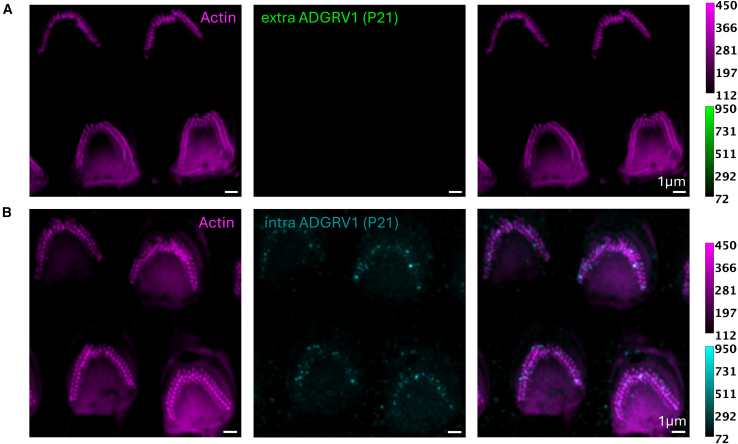
Immunolabeling of ADGRV1 extracellular and intracellular regions in P21 mouse OHCs monitored by two-color STED microscopy Scale bars, 1 μm. The intensity scales are reported for the two colors. (A) Top views of four P21 OHCs STED images with labeling of actin (left, purple), ADGRV1 extracellular region (center, green), and composite image (right). (B) Top views of four P21 OHCs STED images with labeling of actin (left, purple), ADGRV1 intracellular region (center, cyan), and composite image (right).

### P5 IHC labeling

In the mammalian cochlea, the hair bundles of inner hair cells differ greatly in size and shape from those of outer hair cells. IHC hair bundles consist mainly in one tall row (1^st^), one middle row (2^nd^), and a 3^rd^ small row. Their stereocilia rows are flat or slightly curved around the smaller stereocilia. In addition, the maturations of hair cells are different along the tonotopic cochlea’s axis. The hair cell differentiation progresses gradually from the base of the cochlea toward the apex during the first postnatal week.^19^ We have systematically selected hair cells in the middle of the cochlea’s central axis.

#### ADGRV1 extracellular region contributes to ankle links between stereocilia of the 1^st^ row, as well as between stereocilia of 1^st^ and 2^nd^ rows

Qualitatively, top views of ADGRV1 extracellular region labeling in P5 mice IHCs (displayed in green) yields ring-like structures around stereocilia (colored in magenta) of the outer row (Figure 2A). This corresponds to both inter- and intra-row localizations of the receptor. Additionally, the labeling appears granular and discontinuous. No labeling could be observed between stereocilia of the 2^nd^ row. The analysis of the unfolded bundle projection profiles (Figure 2B, right) accordingly highlights two maxima of fluorescence (arrows). One coincides with the position of the stereocilia of the 1^st^ row (any distance coordinate on the profile plot corresponds to a vertical position in the unfolded image), reflecting intra-row labeling of the protein. The second arrow in the profile plot is located away from the 1^st^ stereocilia row at a distance corresponding to about 36% of the inter-row space, corresponding to inter-stereocilia protein labeling. Further analysis based on individual stereocilia superimposition (Figure 2C, left) indeed shows two local maxima in between stereocilia of the 1^st^ row (horizontal profile, center) and one maximum between the two stereocilia rows (vertical profile, right). These results show a contribution of ADGRV1 extracellular region to ankle links between stereocilia of the 1^st^ row, as well as between stereocilia of the 1^st^ and 2^nd^ rows.

In side views of IHCs, labeling of ADGRV1 extracellular region (Figure 2D) highlights a single horizontal band of protein, starting just above the basal end of the stereocilia (taper region) and spanning about 300–400 nm toward the top of the stereocilia. This observation suggests a potential interplay between molecules of the ankle links complex with proteins of the taper, consistently with interactions we have already characterized *in vitro.*^20^

#### ADGRV1 intracellular domain is mostly found within stereocilia of the 1^st^ row

Top views of ADGRV1 intracellular labeling in P5 mice IHCs (displayed in cyan) also yield crown-like patterns around stereocilia of the 1^st^ row (Figure 3A). Similar to the ADGRV1 extracellular labeling, little signal was detected around stereocilia of the 2^nd^ row. Labeling appears, however, qualitatively more continuous in a ring shape close to the stereocilia cores. The analysis of the unfolded bundle projection profiles (Figure 3B, left) highlights two local maxima (Figure 3B, right, arrows). One corresponds again to intra-row labeling of the 1^st^ stereocilia row. The other located about 24% of the inter-row space away from the 1^st^ row, with an additional shouldering at about 62%. This reflects labeling on both sides of the inter-row space. However, as illustrated in the images Figures 3A and 3B, the main contribution to the inter-row space labeling is located near stereocilia of the 1^st^ row, while a minor contribution arises from the 2^nd^ row and mostly near the central kinked segment of the hair bundle. This observation is further confirmed by individual stereocilia superimposition (Figure 3C, left) where only one maximum is observed for ADGRV1 in the vertical profile (right), closer to the 1^st^ row, as that profile does not cross a cilium of the 2^nd^ row. However, the intensity around the 2^nd^ row is significantly weaker. In the horizontal profile (center), two maxima are found in between each stereocilia of the 1^st^ row. Labeling of the cytoplasmic domain allows to conclude on the embedding location of ADGRV1 β subunit. Here, we show that this GPCR moiety of the receptor is mostly found within stereocilia of the 1^st^ row, with less protein embedded within the 2^nd^ row. Our results therefore highlight both symmetrical orientation of ADGRV1 between stereocilia of the 1^st^ row, with molecules embedded in both sides, and asymmetrical organization between rows, with ADGRV1 largely present in the 1^st^ row, while few molecules only arise from the 2^nd^ row.

#### PDZD7 colocalizes with ADGRV1

In juvenile IHCs, labeling of PDZD7 (colored in yellow, Figure 4) qualitatively results in sparse half-circle patterns centered around stereocilia of the 1^st^ row as previously observed with the ADGRV1 intracellular labeling (Figure 4A). This is well illustrated in the unfolded bundle projection profiles, with the typical two maxima observed within the 1^st^ row of stereocilia and away from it at about 35% of the inter-row space (Figure 4B, right). Accordingly, individual stereocilia superimposition highlights two main maxima in the horizontal profile corresponding to intra-row labeling of 1^st^ stereocilia (Figure 4C, center), as well as one maximum in the vertical profile highlighting inter-row labeling (Figure 4C, right). PDZD7 being a cytoplasmic protein, our results indicate that it is mainly present in stereocilia of the 1^st^ row in the hair bundle. As previously reported, PDZD7 is located in the region above the taper, as we observed for ADGRV1, in lateral views of stereocilia.^14^ Therefore, it localizes in stereocilia where ADGRV1β subunit is also found, supporting the interaction and functional interplay of the two proteins *in vivo* in agreement with what has been previously showed in mouse IHCs and OHCs.^14^^,^^21^ This observation is also consistent with our results showing a tight interaction *in vitro* between the last few cytoplasmic residues of ADGRV1 and the N-terminal region of PDZD7.^10^

### P5 OHC labeling

The hair bundles of OHCs consist of three vertical rows of stereocilia—1^st^ (the tallest), 2^nd^ and 3^rd^—arranged in a staircase pattern, forming a U shape curved around the smaller stereocilia.

#### ADGRV1 extracellular region is exclusively observed in the inter-row spaces between stereocilia

Qualitatively, labeling of ADGRV1 extracellular region in P5 OHCs yields a two-line pattern in top views, filling both inter-row spaces between 1^st^ and 2^nd^ stereocilia and between 2^nd^ and 3^rd^ stereocilia (Figure 5A). Analysis of the unfolded bundle projection unambiguously highlights two maxima in between stereocilia rows (Figure 5B, arrows). In addition, the plot of the horizontal profile of the averaged stereocilia of the 1^st^ row does not show any significant signal (Figure 5C, center), neither does the profile of the averaged stereocilia of the 2^nd^ row (Figure 5D, center), reflecting the absence of labeling within stereocilia rows. In the vertical profiles, maxima are observed in the inter-row space (right panels of Figures 5C and 5D). Labeling of ADGRV1 extracellular region in juvenile OHCs therefore yields different patterns within the hair bundle compared to IHCs. In OHCs, labeling is exclusively observed in the inter-row spaces and not between stereocilia of a same row. We noted the absence of labeling between the stereocilia of the same row, whatever the plane that we analyzed along the main axis of the stereocilia (Figure S5). This result also indicates that ADGRV1 molecules have to be embedded in stereocilia of either the 2^nd^ and/or 3^rd^ row of stereocilia in addition to the 1^st^ row during the hair bundle development.

A partially tilted view of an OHC with ADGRV1 extracellular region labeling highlights one band of signal (Figure 5E), also observed with ADGRV1 extracellular region labeling in IHC (Figure 2D). Qualitatively, it appears that labeling of ADGRV1 extracellular region is also located only just above the taper region in OHCs. More quantitatively, we can estimate from Figure S5, a z stack movie (in depth, steps 300 nm) of OHC hair bundles in top view that the distance between ADGRV1 and the cuticular plate lies between 300 nm and 600 nm and the distance to the tip of the stereocilia lies between 1.2 and 1.5 μm.

#### The β subunit of ADGRV1 is found within stereocilia of the two main rows

Labeling of ADGRV1 cytoplasmic domain in top viewed OHCs yields more structured patterns compared to the distribution of the extracellular region (Figure 6A). Still, a close look at the merged selected image indicates that labeling is tied to the inter-row space. Using our unfolding method of the hair bundle image, we unambiguously highlight three maxima (Figure 6B, right, arrows). The first two are located on each side of the space between the 1^st^ and 2^nd^ row. This indicates that the β subunit of ADGRV1 is found within stereocilia of the two rows, resulting in a symmetrical contribution of the receptor to inter-row ankle links in OHCs. The third unambiguous maximum is located in the space between the 2^nd^ and 3^rd^ row of stereocilia, close to the 2^nd^ row. This indicates that ADGRV1 molecules embedded in the 2^nd^ row are oriented toward either the 1^st^ or the 3^rd^ stereocilia row. However, it is unclear whether ADGRV1 locates to stereocilia of the 3^rd^ row, as labeling is barely observed in our dataset independently of the chosen focus. Finally, the horizontal intensity profile derived from individual stereocilia superimposition confirms the absence of labeling between stereocilia of a same row (Figures 6C and 6D, center), while the vertical intensity profile highlights again the symmetrical distribution of ADGRV1 on both sides of the inter-row space (Figure 6C, right).

#### PDZD7 is expressed in stereocilia of every row such as ADGRV1

In OHCs, PDZD7 labeling is characterized by a similar distribution as ADGRV1 cytoplasmic domain (Figure 7). The intensity of labeling is weak, but our images qualitatively suggest that PDZD7 is expressed in stereocilia of the 1^st^ and 2^nd^ rows in OHCs (Figure 7B, right). Again, this is consistent with PDZD7 being addressed in the same cellular environment as the ADGRV1β subunit.

Representative average distributions of ADGRV1 (extra- and intra-cellular regions) and PDZD7 at P5 (average projections of aligned single stereocilia of the 1^st^ row of IHCs and OHCs) are shown in Figure 8 for easier comparison.

### P17/P21 OHC labeling

As mentioned in the introduction, ADGRV1, usherin, PDZD7, and whirlin are transiently expressed in hair cells during the development. In mouse cochlear hair cells, the stereocilia ankle-links are detected between P2 and P12, while the inner ear is considered fully matured at P25. Therefore, we expect no labeling of ADGRV1 extracellular region to be observed after P15. However, no information is available regarding a potential hydrolysis of ADGRV1 α and β subunits, potentially allowing for a maintained GPCR moiety of the protein after disappearance of the ankle links. To challenge that hypothesis, we have imaged the extracellular region and cytoplasmic domain of ADGRV1 at P17, respecting both the exact same labeling protocol and same imaging conditions. As expected, we did not detect any labeling of ADGRV1 extracellular region in P17 mice OHCs (Figure 9A), which is consistent with the disappearance of the ankle links reported in the literature. However, we observed the unambiguous sparse presence of ADGRV1 cytoplasmic domain in P17 mice OHCs (Figure 9B). The sets of ADGRV1 images use the same normalization. A similar analysis has been performed at P21, which confirms the initial hypothesis that the extracellular ADGRV1 is absent at the latest after P17 (Figure 10A) and that ADGRV1β is detected in OHCs after the hair cell development up to P21 (Figure 10B). Finally, we could not acquire images of sufficient quality to draw conclusions about ADGRV1β in IHCs.

## Discussion

ADGRV1 is a main component of the transient ankle links, connecting stereocilia of the developing cochlear hair cells.^6^^,^^7^ The receptor is roughly collocated with the other members of the ankle-link molecular complex in the basal region of the stereocilia. By using super resolution light microscopy on a whole mouse cochlea, we have localized ADGRV1 and PDZD7 in hair bundles at an unprecedented level of precision at different developmental stages, i.e., P5, P17, and P21. STED super-resolution fluorescence imaging, unlike confocal imaging, allowed to characterize the intra- and inter-row localization of the two proteins. To differentially monitor the localization of extracellular and intracellular regions of ADGRV1, we benefited from two different antibodies specifically directed against the extracellular region of ADGRV1 (subunit α) or against its cytoplasmic domain (subunit β), respectively.

**Figure 11:**
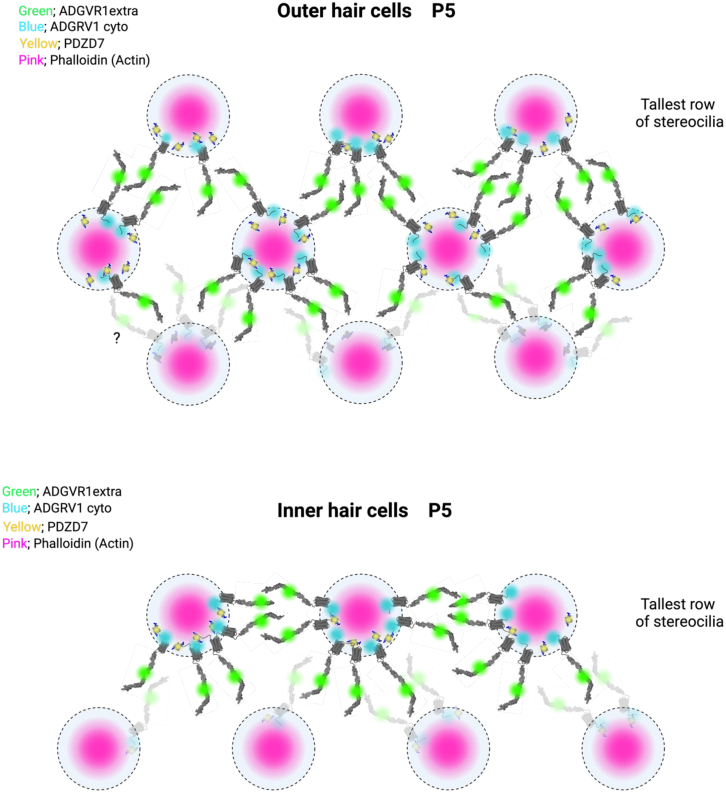
Schematic representation of ankle links in OHCs and IHCs Top views of OHCs (top) and IHCs (bottom). Actin is colored in purple, ADGRV1 extracellular epitope in green, ADGRV1 intracellular epitope in blue, PDZD7 in yellow. Delimitations of stereocilia are indicated by dashed circles. Created with BioRender.com.

We revealed that ADGRV1 is differentially expressed in IHC and OHC hair bundles during the hair cell development (Figure 8), showing respectively asymmetrical (IHC) and symmetrical (OHC) distribution. In IHCs, ADGRV1 is mainly observed in stereocilia of the 1^st^ row, with its extracellular region oriented toward stereocilia of the 1^st^ and 2^nd^ rows. This results in an asymmetrical distribution across the inter-row space. The same localization is observed for the intracellular region of ADGRV1, suggesting that we have detected the full-length isoform of ADGRV1, mainly present in the 1^st^ row and weakly found in the 2^nd^ row at P5. In OHCs, the intracellular domain of ADGRV1 is localized at the base of stereocilia in all rows, while the extracellular region is directed exclusively toward stereocilia of adjacent rows with no intra-row labeling. Despite the high resolution of our STED images, we were unable to unambiguously conclude whether the symmetrical inter-row distribution of the GPCR in adjacent stereocilia results in crossing extracellular regions stemming from both sides or if only one stereocilia contributes the full-length protein, while the other encompasses solely the β subunit. On the other hand, given the clear embedding of the receptor on each side, we propose that the neighboring stereocilia provide ADGRV1 molecules to form the fibrous links (Figure 11).

The difference in ADGRV1 distribution among hair cells may suggest distinct functional roles of ADGRV1 within the two sensory cell types. It has been previously shown that the absence of ADGRV1 expression in mice leads to different morphology of OHCs and IHCs. At early postnatal days (P0–P2), hair bundle morphology in wild-type and ADGRV1 mutant mice are mostly indistinguishable while clear changes appear in mutant mice by P4,^6^ for which OHCs exhibit a U-shaped configuration contrasting with the V shape morphology observed in wild-type specimens, while IHCs display asymmetrical hair bundle arrangements instead of the symmetrical arch-shaped structures typically observed in wild-type animals.^7^ An additional role of ADGRV1 is to inhibit the development of a peripheral subpopulation of microvilli, mainly localized at the neural edge side of the inner hair cells. These microvilli could also affect the shape of the hair bundle.

At the same time, we observed a spatial distribution for PDZD7 similar to that of ADGRV1β in both IHCs and OHCs at P5 (Figure 8), in agreement with previously published results showing, by confocal microscopy imaging, a co-localization at a lower resolution of both proteins at the base of the stereocilia and their tight interaction *in vitro.*^10^^,^^14^^,^^22^ It has been also shown that PDZD7 is essential for the subcellular localization of the three USH2 proteins ADGRV1, usherin, and whirlin at the ankle link position in both IHCs and OHCs through the study of PDZD7 knock-out mice,^14^^,^^23^ illustrating its function as a primary scaffold for the complex assembly of the Usher 2 proteins. To note, the deletion of the PDZD7 long isoform strongly affects the OHC stereocilia development; as observed for the ADGRV1 mutant mice, the hair bundles of OHCs lost its usual V-shaped symmetry.^23^ At P4, abnormal morphologies in Pdz7^−/−^ mice were observed with missing stereocilia and bent hair bundles with stereocilia of atypical length and thickness. On the contrary, IHCs are mostly unaffected. We propose that the inherent differences in the shape and organization of hair bundles in OHCs and IHCs could at least partially result from the difference in ankle link distribution and polarity between stereocilia that we monitored in this study in developing cochlear hair cells.

The morphological organization of hair bundles is critical for sensitivity and frequency selectivity to sound-induced vibrations. The proper development and maintenance of conserved architecture of hair bundles suggest a finely regulated expression of proteins, likely through cell signaling pathways. However, few proteins have been identified to initiate such signaling. ADGRV1, a GPCR, is a key candidate.^24^ We demonstrate here that the GPCR domain of ADGRV1 is detected at later stages of hair cell development in mice, consistent with a possible signaling turning point around P12, mediated by the dissociation of ADGRV1’s extracellular region. The GAIN domain, located just upstream of the first transmembrane helix, is necessary and sufficient for the adhesion GPCR autoproteolysis.^25^^,^^26^ ADGRV1 is thus thought to undergo autoproteolysis of its N-terminal region, parting the α (the extracellular region) and β (the GPCR and cytoplasmic domains) subunits. The new N-terminal of the GPCR forms an agonist Stachel peptide that may stay bound to the GAIN domain via hydrogen bonds in a non-covalent heterodimer ADGRV1α and ADGRV1β.^9^^,^^27^ Full-length ADGRV1 could be exposed on stereocilia as a cleaved, yet associated protein. *In vitro*, ADGRV1 associates with Gs/Gq proteins in its undissociated form.^9^^,^^24^^,^^28^ Spontaneous or external stimulus-driven (mechanical stress, calcium variation, …) dissociation of the GAIN domain may free the Stachel peptide, activating the protein and promoting different G protein coupling, as shown for other adhesion GPCRs.^29^ After dissociation, ADGRV1 may be activated in a Stachel-mediated manner and coupled to Gi proteins.^9^ At P5, both ADGRV1α and β subunits are detected at the base of stereocilia with similar distributions, strongly suggesting the presence of full-length ADGRV1. It is still unclear whether dissociation of the subunits precedes ankle-link disappearance, as both may coexist independently at this stage. In contrast, we show that ADGRV1β is maintained until P21 in mice, while ADGRV1α disappears around P12, consistent with ankle-link loss. Therefore, the GPCR domain of ADGRV1 is likely separated from the extracellular region around P12, becoming active and possibly initiating signaling through the Gi pathway, as suggested *in vitro.*^9^ At later stages (P17 and P21), the negative regulator of ADGRV1, PDZD7, was weakly detected in hair cell stereocilia in our STED images, aligning with previous findings in adult cochlea.^14^ Recent proteomic studies suggest that long and short isoforms of ADGRV1 in cellular models can regulate several pathways, including AMPK, MAPK, Notch, Ephrin, and Wnt signaling, as well as cellular Ca^2+^ homeostasis, transcriptional regulation, cell differentiation, and ciliary integrity.^24^ However, the consequences in terms of signaling in hair cells, such as gene expression, have not been yet documented.

The study at super resolution of ADGRV1 and PDZD7 within hair bundles has revealed their fine localizations between and along the stereocilia. The distribution patterns of both proteins are different in IHCs and OHCs and between the different stereocilia rows of hair bundles. Moreover, ADGRV1 and PDZD7 are not only preferentially localized in certain rows of the hair bundle but also show an asymmetrical pattern around the main axis of stereocilia. The regulatory mechanisms responsible for the remarkably restricted localization of ADGRV1 and PDZD7 are not yet fully understood. However, these mechanisms must ensure the precise cellular addressing of ADGRV1 and PDZD7, in coordination with other components of the ankle link complex, to specific stereocilia during development of the cochlear hair bundles.

### Limitations of the study

While this work provides insights into the spatial organization of ADGRV1 and PDZD7 in developing auditory hair cells, some limitations should be considered. The analysis focused on selected developmental stages (P5, P17, and P21), offering only a partial view of the dynamic changes these proteins undergo during cochlear maturation. As a result, the exact timeline and mechanisms underlying the proposed dissociation of ADGRV1 subunits remain unclear. Additionally, although STED microscopy provided nanoscale resolution, the use of conventional immunolabelling in this study induces a non-negligible uncertainty regarding the localization of the proteins of interest. Therefore, it remains difficult to determine whether the observed inter-stereocilia signals result from symmetric contributions from adjacent rows or represent an asymmetric distribution originating from a single row. Finally, this study was descriptive in nature and did not include functional experiments to directly test how the spatial arrangement of these proteins influences hair bundle morphology or signaling. To further complete this study, a high-resolution mapping of the two other proteins of the Usher2 complex, usherin and whirlin, would need to be investigated.

## Resource availability

### Lead contact

Further information and requests for resources and reagents should be directed to and will be fulfilled by the lead contact, Nicolas Wolff (nicolas.wolff@pasteur.fr).

### Materials availability

This study did not generate new unique reagents. All requests for resources and reagents should be directed to and will be fulfilled by the lead contact.

### Data and code availability

• The sources of the datasets supporting the current study are presented in the key resources table and the method details sections.
• Requests for the data and code that support the findings of this study should be directed to the lead contact. The macro, written in Fiji, for interactive and user-friendly analysis of STED images of hair bundles of this study is publicly available via the following link: https://gitlab.pasteur.fr/moneron/fiji_hair_cells. Raw data are available from lead contact upon reasonable request.
• All other items: any additional information needed for data presented in this paper is available from the lead contact upon request.

## Acknowledgments

We thank Dr. M. Sato (Osaka University, Japan) for sharing *Adgrv1*^*t*m1Msat^ mice. We thank Pr Petit (Institut de l’Audition, Institut Pasteur, France) for discussions and advice. The authors thank J.P. Corringer for discussions and critical reading of the manuscript. We are grateful to N. Barilone for her support in the immunolabeling experiments of HEK cells. This work was supported by an Institut Pasteur Axe 3 seed grant to G.M. and N.W., the 10.13039/100019671Fondation Pour l'Audition (program N°2019-0018C1) to N.W., the Ministère de l’Enseignement Supérieur et de la Recherche (grant N°3178/2018) to B.C.-C., and the 10.13039/501100002915Fondation pour la Recherche Médicale (grant N°FDT202106013076) to B.C.-C. The project has received funding from the European Union's Horizon 2022 research and innovation program under the Marie Slokdowska-Curie grant agreement CLEXM N°101120151. This work was also supported by a grant from Région Ile-de-France (DIM ELICIT 2019) to G.M. for the STED microscope, co-funded by 10.13039/501100003762Institut Pasteur.

## Author contributions

B.C.C., G.M., A.E.H., V.M., and N.W. conceptualized and designed the study. B.C.C., G.M., A.E.H., V.M., and N.W. supervised the study. B.C.C., G.M., A.E.H., V.M., and N.W. conducted the experiments. B.C.C., G.M., A.E.H., V.M., and N.W. analyzed and interpreted the data. B.C.C., G.M., A.E.H., D.D., V.M., and N.W. wrote, reviewed, and edited the manuscript. All authors read and approved the final manuscript.

## Declaration of interests

The authors declare no competing interests.

## STAR★Methods

### Key resources table

**Table:** 

REAGENT or RESOURCE	SOURCE	IDENTIFIER
**Antibodies**		

PDZD7 Rabbit Polyclonal Antibody – C terminal	My BioSource	MBS3217600; Lot# QC38272-41164
Extracellular ADGRV1 Rabbit Polyclonal antibody	Invitrogen GPR98	GPR98; #PA5-84761; RRID: AB_2791911
Rabbit antibodies against ADGRV1 cytoplasmic domain	Agro-Bio	N/A
Secondary Rabbit IgG antibody coupled to an STAR RED dye	Abberior	STRED-1002; RRID: AB_2833015

**Chemicals, peptides, and recombinant proteins**		

Phalloidin- Alexa 594	Invitrogen	A12381
STAR Orange dye	Abberior	STORANGE-1002
Mowiol/DABCO mounting medium	Calbiochem, Darmstadt, Germany, Wurm et al.,^30^ 2010.	Mowiol 4–88
DMEM 1X	Gibco	31966–021
BSA	Sigma	A6003
FBS	Gibco	A5670701
NGS	Abcam	AB7481
PBS Buffer 10X Concentrate	Interchim	N14013
Triton X-100 - BioXtra	Sigma	T89284
Paraformaldehyde solution 4% in PBS	ChemCruz	SC-281692

**Critical commercial assays**		

JetPRIME	Polyplus	#101000015
Lipofectamine 3000	Invitrogen	L3000-008

**Experimental models: Cell lines**		

HEK293T cells	ATCC	N/A

**Experimental models: Organisms/strains**		

Mouse: OF1	Charles River	N/A
Mouse: B6CBAF1/J	The Jackson Laboratory	N/A
Mouse: C57BL/6J	The Jackson Laboratory	N/A
Mouse: Adgrv1tm1Msat (Adgrv1^+/−^ and Adgrv1^−/−^)	Dr. Yagi, Hyogo Medical University, and Pr. Sato, University of Osaka, Japan	N/A

**Recombinant DNA**		

pMT3-GFP	This paper	N/A
pMT3-GFP-PDZD7	This paper	N/A

**Software and algorithms**		

ImageJ2	Rueden et al.^31^	https://imagej.nih.gov/ij
Imspector Image Acquisition Software	Abberior Instruments Development Team	http://www.imspector.de

**Deposited data**		

Fiji Analysis Macro **fiji_hair_cells**	This paper	https://gitlab.pasteur.fr/moneron/fiji_hair_cells

### Experimental model and study participant details

#### Animal handling

All animals were OF1 mice provided by Charles River, and B6CBAF1/J, C57BL/6J mice provided by the Animal facility of Institut Pasteur, Paris, France. Juvenile mice were sacrificed at postnatal day 5 by decapitation and at postnatal days 17, 21 by CO2 inhalation. Animal experiments were carried out in accordance with European Community Council Directive 2010/63/UE under authorizations CETEA N°200007 from the Institut Pasteur ethics committee for animal experimentation. A total of 228 cochleae from 114 mice were used in this study, including optimization. Males and females were used indifferently. Cochleae were examined at the following mice ages: P5, P17 and P21. *Adgrv1*^tm1Msat^ (*Adgrv1*^+/−^ and *Adgrv1*^−/−^) mice were kindly provided by Dr. Yagi, Hyogo Medical University, and Pr. Sato, University of Osaka, Japan.^17^^,^^18^

#### Cell culture conditions

HEK-293T cells were cultured in DMEM supplemented with 10% FBS and 1% penicillin-streptomycin at 37°C in a humidified incubator with 5% CO_2_.

### Method details

#### Immunofluorescence of HEK cells

Sterile lamellae were placed in a 24-well plate, coated with 0.01% poly-L-lysine solution for 30 min at room temperature, then rinsed three times with sterile PBS and allowed to dry before cell seeding. HEK-293T cells were seeded at 70% confluency onto poly-L-lysine-coated lamella and transfected using Lipofectamine 3000 according to the manufacturer’s protocol. A transfection mixture containing Lipofectamine 3000 reagent, P3000 reagent, and plasmid DNA (pMT3-GFP and pMT3-GFP-PDZD7) was prepared in Opti-MEM, incubated for 15 min at room temperature, and then added dropwise to the cells. After 48 h, cells were subjected to 48 h before immunofluorescence staining. Following transfection, cells were washed three times with PBS and fixed with 4% paraformaldehyde (PFA) for 15 min at room temperature, followed by three additional times with PBS. Permeabilization was performed with 0.1% Triton X-100 in PBS for 10 min, followed by blocking of non-specific binding with 10% bovine serum albumin (BSA) in PBS for 1 h at room temperature. Cells were then incubated overnight at 4°C with PDZD7 Rabbit Polyclonal Antibody – C-terminal (MyBioSource #MBS3217600) diluted in 1% BSA/PBS. After three PBS washes, cells were incubated with Goat anti-rabbit IgG STAR RED (Abberior STRED) at 1/500 for 1 h at room temperature in the dark. After three additional PBS washes, lamellae were carefully transferred onto glass slides with FluoroShield mounting medium containing DAPI. Fluorescent images were acquired using a confocal microscope with optimized laser settings and processed using ImageJ Fiji.

#### Sample preparation

At the three ages of mice (P5, P17 and P21), the cochleae were extracted in PBS, then fixed in PBS containing 4% paraformaldehyde (PFA). The fine dissection of the organ of Corti was performed and the sample was stored it at 4°C until use. Whole organs were washed in PBS before blocking and permeabilization using PBS supplemented with 20% nature goat serum and 0.1% Triton X-100 for 30 min. Incubation of the primary antibodies was performed overnight in PBS BSA 4%: rabbit anti-ADGRV1 extra 1/100 (*Invitrogen* GPR98 Polyclonal antibody, #PA5-84761), rabbit anti-PDZD7 1/100 (*MyBioSource* antibodies PDZD7 polyclonal antibody, #MBS3217600). Rabbit antibodies against ADGRV1 cytoplasmic domain were raised against the C-terminal 152 residues of the protein purified from *E. coli extract* and used at a concentration of 1.4 mg/mL (concentration source) (1/100 purified) (*Agro-Bio*). In double-labeling experiments for both ADGRV1 and actin imaging, after washing with PBS, a commercial secondary anti-Rabbit IgG antibody coupled to an STAR RED dye (*Abberior*, #STRED-1002) diluted to 5 μg/mL in PBS +4% BSA, as well as 3 units (∼0.5 mM) of phalloidin coupled to an Alexa 594 or STAR Orange dye (*Invitrogen*, #A12381; *Abberior*, #STORANGE-1002) were added to the sample and incubated for 2 to 4 h at 4°C. Whole organs of Corti were then washed with PBS and mounted on regular microscopy slides with Mowiol/DABCO mounting medium^30^ and topped with 18 mm diameter N^o^ 1.5H cover-slips (*Marienfeld*, #0117580).

#### Image acquisition

Super-resolution fluorescence microscopy was performed on a commercial STimulated Emission Depletion microscope (STED expert line – Abberior Instruments) using an Olympus 100X/1.4 NA oil objective lens. Two color STED images have been obtained in line interleaved mode, switching on one excitation laser line at a time, 560 nm for STAR Orange or Alexa 594 and 640 nm for STAR RED. The laser line for STED was 775 nm - pulsed. Pixel sizes were between 10 nm and 25 nm and elementary pixel dwell times of 1μs with up to a few tens of line averages, depending on the acquisitions.

#### Image analysis

Analysis of the super-resolution STED images was performed using the open-source software *Fiji*.^32^ All images were analyzed with the same interactive workflow, bundled into a single macro written specifically for this analysis. The results of all the steps of the workflow and for each analysis were automatically saved as independent intermediate files. The first step of the macro consists in opening independently the raw color channels, the image of actin of the cilia on one side and the image of the distribution of the protein of interest on the other side (ADGRV1 or PDZD7). The user is then asked to manually select, roughly with a brush of the appropriate predefined size, the row of cilia to take into account (in the image of actin). A mask is generated to define a first region of interest on which a Gaussian blur filter with a standard deviation of 2 pixels is applied to smooth out the pixel noise. In that region of interest, the built-in detection of local maxima function “Find maxima” is applied to find the centers of the cilia, for which coordinates are saved in a table. The blurred version of the masked initial image is then closed, and all subsequent operations are done on raw pixels. The user can visualize the detected cilia and can decide to exclude undesired maxima from a first selection, typically the ones resulting from a too large definition of the initial region of interest. After validation of the selection by the user, selected maxima are automatically reordered and saved in a new table of coordinates to start from one extremity of the hair bundle and such that consecutive elements correspond to the closest neighbors. This order enables to create a spline curve passing through the center of the cilia. In order to have a representation of the average profile of the distribution of the protein of interest, relatively to the cilia, across the selected row of cilia, two unfolded linear images along the spline curve are created, using the built-in function “straighten”; one of a row of cilia and one of the protein distribution by applying the same deformation of space to both initial raw images. Orthogonal averaged profiles of the two unfolded images are then easily extracted and overlaid in a single figure. The averaged profile of each channel is normalized to its maximum and the origin of distances corresponds to the position of the spline curve passing through the maxima of the cilia.

To produce another representation of the average distribution of the protein of interest around cilia of the selected row, a superimposition of individual cilia and surrounding protein is computed. The first step of this second analysis calculates, for each cilium of the selected list, the vector locally orthogonal to the row of cilia, pointing toward the outside of the hair bundle, based on the identification of the circle passing through the considered cilium and its two closest neighbors. At that point, the macro gives the user the opportunity to select cilia to exclude from the projection (any obviously non representative single cilium). Based on the normal vector previously calculated, the local angular orientation of each cilium is calculated and used to copy, rotate a region of interest, and paste it in a stack such that all individual cilia in the stack have a common vertical normal vector. The size of the cropped regions of interest is arbitrarily chosen to be twice the inter-cilia distance, and such as the averaged single cilium is placed in the center with parts of its closest neighbors on both sides. The equivalent stack of the distribution of the protein of interest is created in parallel using the same coordinates and angular information. From the two stacks relative to individual cilia, averages for the two channels are produced. The lookup tables of the results are then adapted for optimal representation by adjusting the respective maxima to the full scales and by subtracting respective backgrounds in the projections according to one of the 3 proposed options. The first calculates the background to subtract by measuring the average of an automatically predefined sub-region of interest where only background is expected, typically in the upper quarter of the projection, outside of the hair bundle. The second option enables the user to select the region of interest supposed to represent the background only. The third option enables to recall the value of the background to take into account from a previous analysis of acquisitions performed in comparable conditions. This last option is particularly useful for the normalization of control conditions in which the protein of interest is supposed to be absent from the preparation. Horizontal and vertical profiles across the average central cilium are subsequently plotted, corresponding respectively to the intra- and inter-row of cilia.

## Supplemental information

Document S1. Figures S1–S4

Figure S5. Movie of a depth stack of ADGRV1 extracellular region and actin in P5 mouse OHCs monitored by two-color STED microscopy (300 nm steps in depth). Actin colored in purple and the extracellular region of ADGRV1 in green.
