# Supplementary material for: Super-resolution mapping of the ankle link proteins ADGRV1 and PDZD7 in developing auditory hair cells
Source: iScience. 2025 Jul 24;28(8):113190. doi: 10.1016/j.isci.2025.113190 (PMC12361621; doi:10.1016/j.isci.2025.113190)
Supplement: Document S1. Figures S1–S4 [file mmc1.pdf]

iScience, Volume *28*

Supplemental information

Super-resolution mapping of the ankle link proteins ADGRV1 and PDZD7 in developing auditory hair cells

Baptiste Colcombet-Cazenave, Gaël Moneron, Antonio El Helou, David DiGregorio, Vincent Michel, and Nicolas Wolff

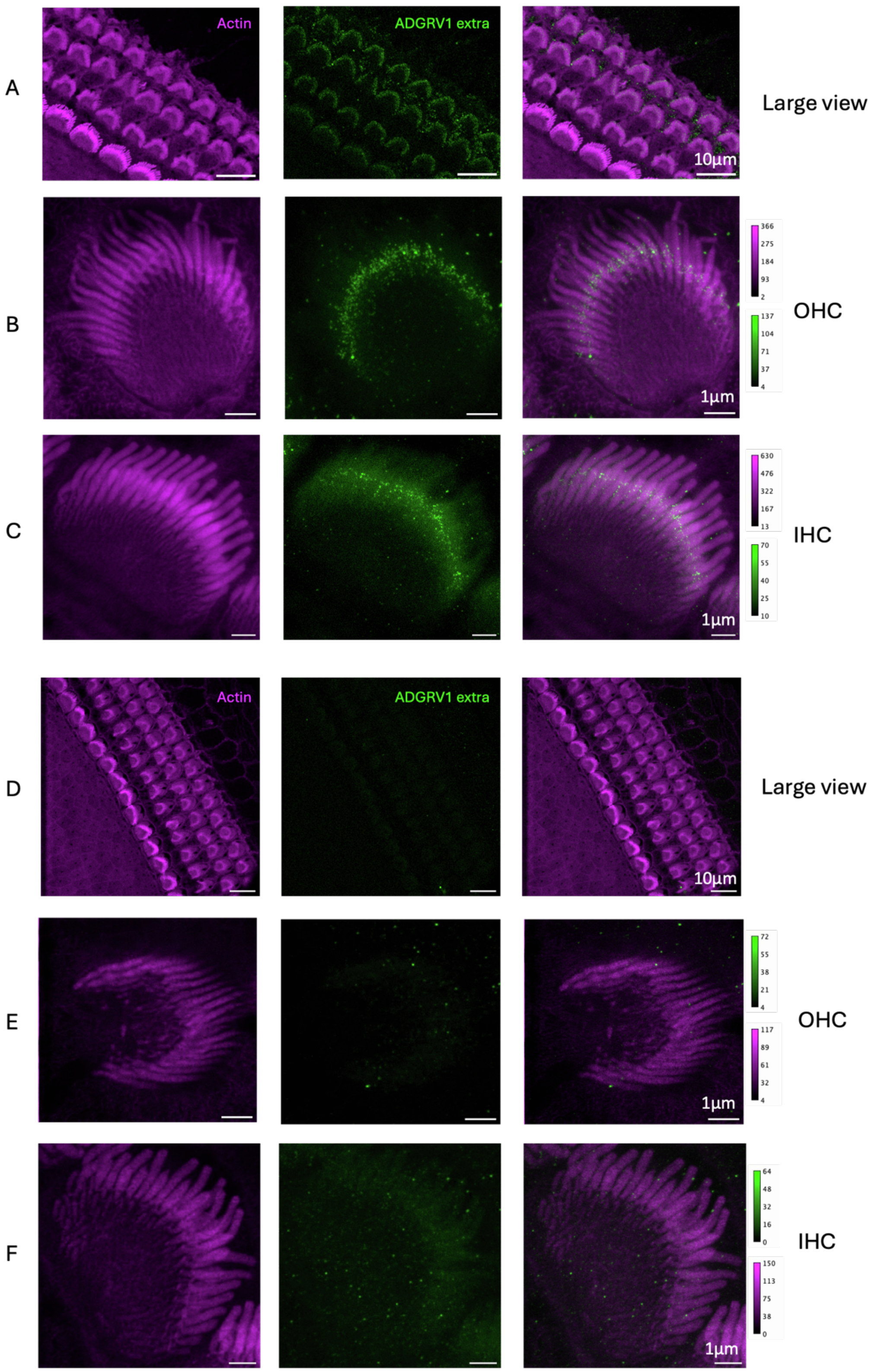
A
B
C
D
E
F
Actin
ADGRV1 extra
Large view
OHC
IHC
10µm
1µm

Figure S1. Immunolabeling of ADGRV1 extracellular region in P5 *Adgrv1*$^{+/+}$ and *Adgrv1*$^{-/-}$ mice, related to figures 2 and 5

Two-color STED microscopy imaging of actin and ADGRV1 extracellular region in P5 *Adgrv1*$^{+/+}$ and *Adgrv1*$^{-/-}$. Scale bar, 1µm. The intensity scales are reported for the two colors. Labeling of actin (left, purple), extracellular region of ADGRV1 (center, green), and the composite image (right).

(A) Overview of hair cell rows in cochlea from *Adgrv1*$^{+/+}$ mouse.

(B) Lateral view of one OHC in cochlea from *Adgrv1*$^{+/+}$ mouse.

(C) Lateral view of one IHC in cochlea from *Adgrv1*$^{+/+}$ mouse.

(D) Overview of hair cell rows in cochlea from *Adgrv1*$^{-/-}$ mouse.

(E) Lateral view of one IHC in cochlea from *Adgrv1*$^{-/-}$ mouse.

(F) Lateral view of one IHC in cochlea from *Adgrv1*$^{-/-}$ mouse.

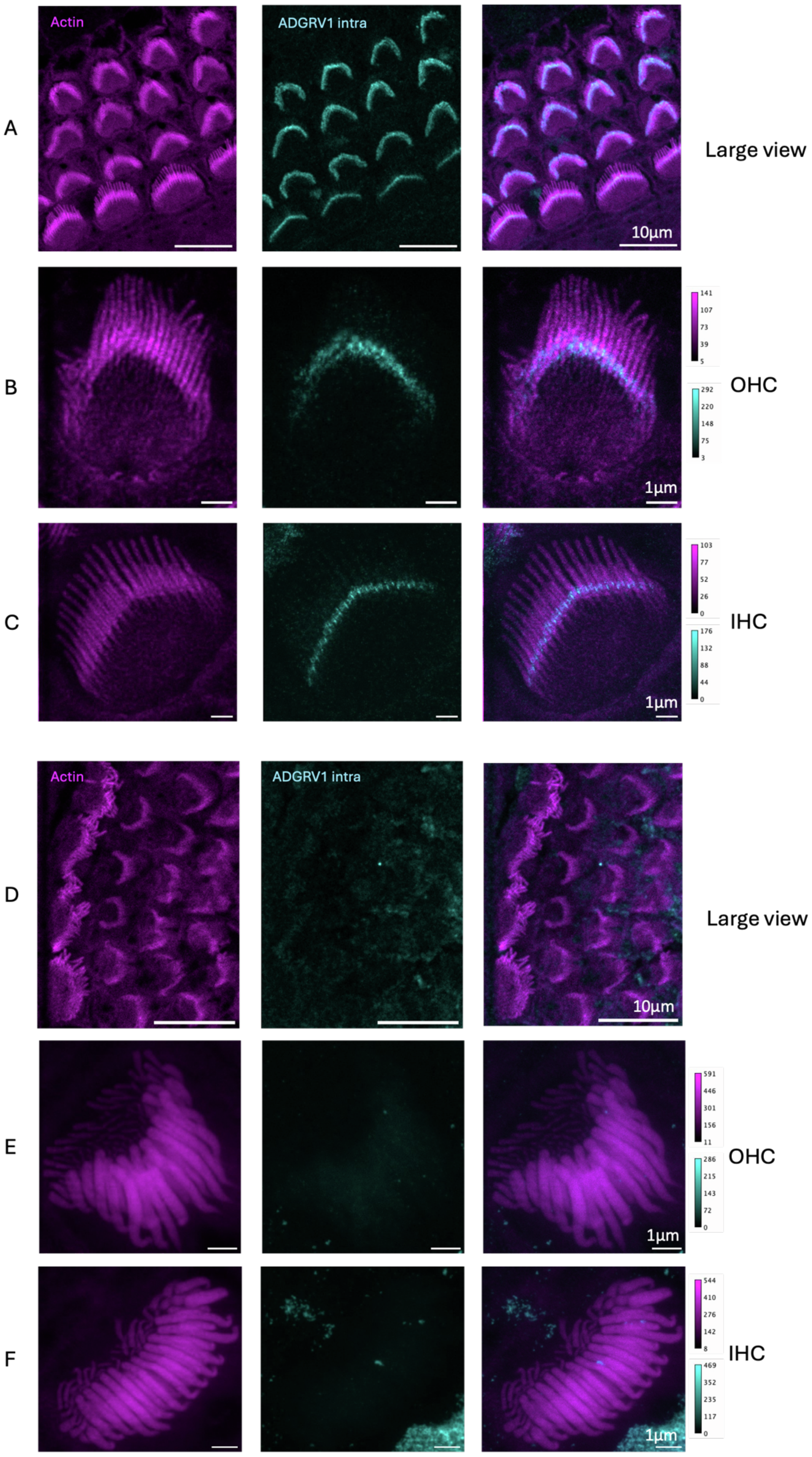

A
B
C
D
E
F
Actin
ADGRV1 intra
Large view
OHC
IHC
10µm
1µm

Figure S2. Immunolabeling of ADGRV1 intracellular region in P5 *Adgrv1*$^{+/+}$ and *Adgrv1*$^{-/-}$ mice, related to figures 3 and 6

Two-color STED microscopy imaging of actin and ADGRV1 intracellular region in P5 *Adgrv1*$^{+/+}$ and *Adgrv1*$^{-/-}$. Scale bar, 1µm. The intensity scales are reported for the two colors. Labeling of actin (left, purple), intracellular region of ADGRV1 (center, cyan), and the composite image (right).

(A) Overview of hair cell rows in cochlea from *Adgrv1*$^{+/+}$ mouse.

(B) Lateral view of one OHC in cochlea from *Adgrv1*$^{+/+}$ mouse.

(C) Lateral view of one IHC in cochlea from *Adgrv1*$^{+/+}$ mouse.

(D) Overview of hair cell rows in cochlea from *Adgrv1*$^{-/-}$ mouse.

(E) Lateral view of one IHC in cochlea from *Adgrv1*$^{-/-}$ mouse.

(F) Lateral view of one IHC in cochlea from *Adgrv1*$^{-/-}$ mouse.

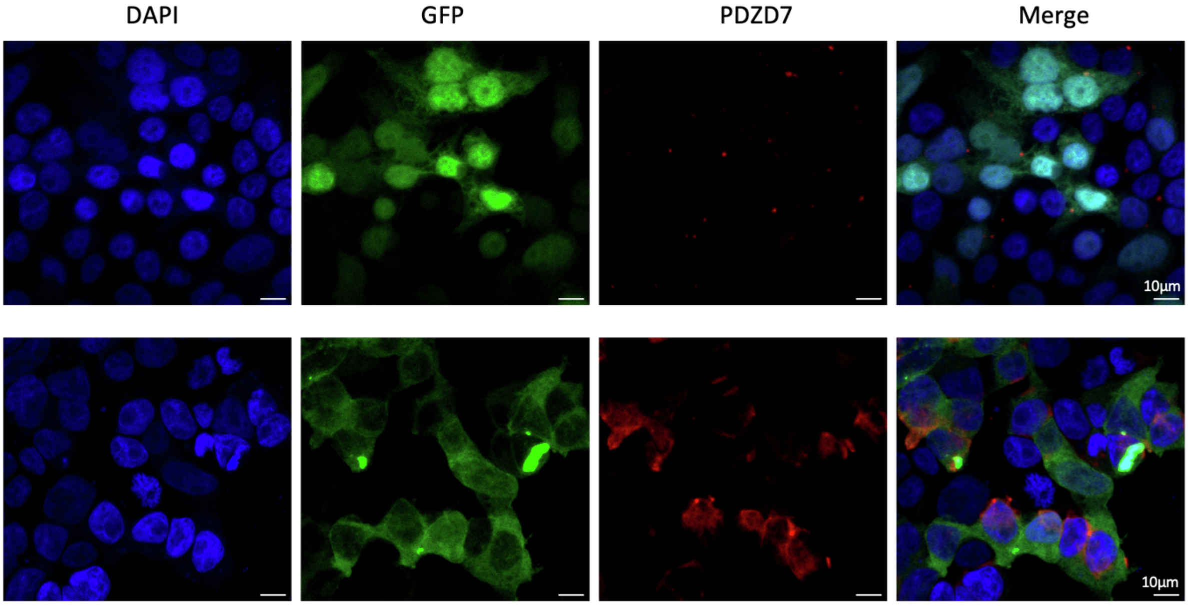

Figure S3. Immunolabeling of PDZD7 in HEK cells, related to figures 4 and 7
Confocal microscopy imaging of HEK cells expressing either the GFP protein (upper panel) or a fusion GFP-PDZ protein (lower panel). Labeling of nuclei (1st column, blue), GFP (2nd column, green), PDZD7 (3rd column, red) and the composite image (4th column). Scale bar, 10µm.

A IHCs ADGRV1 extra

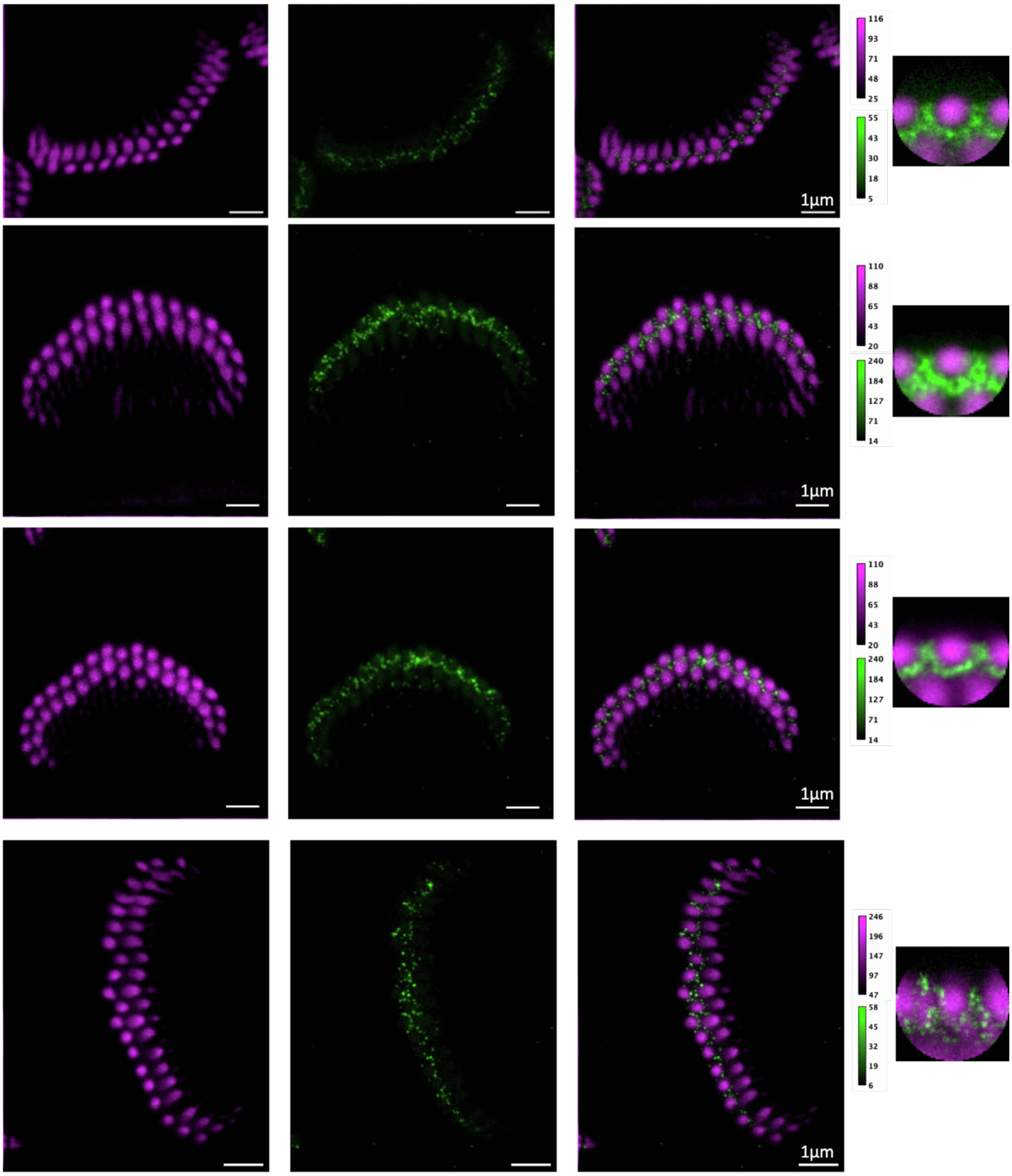

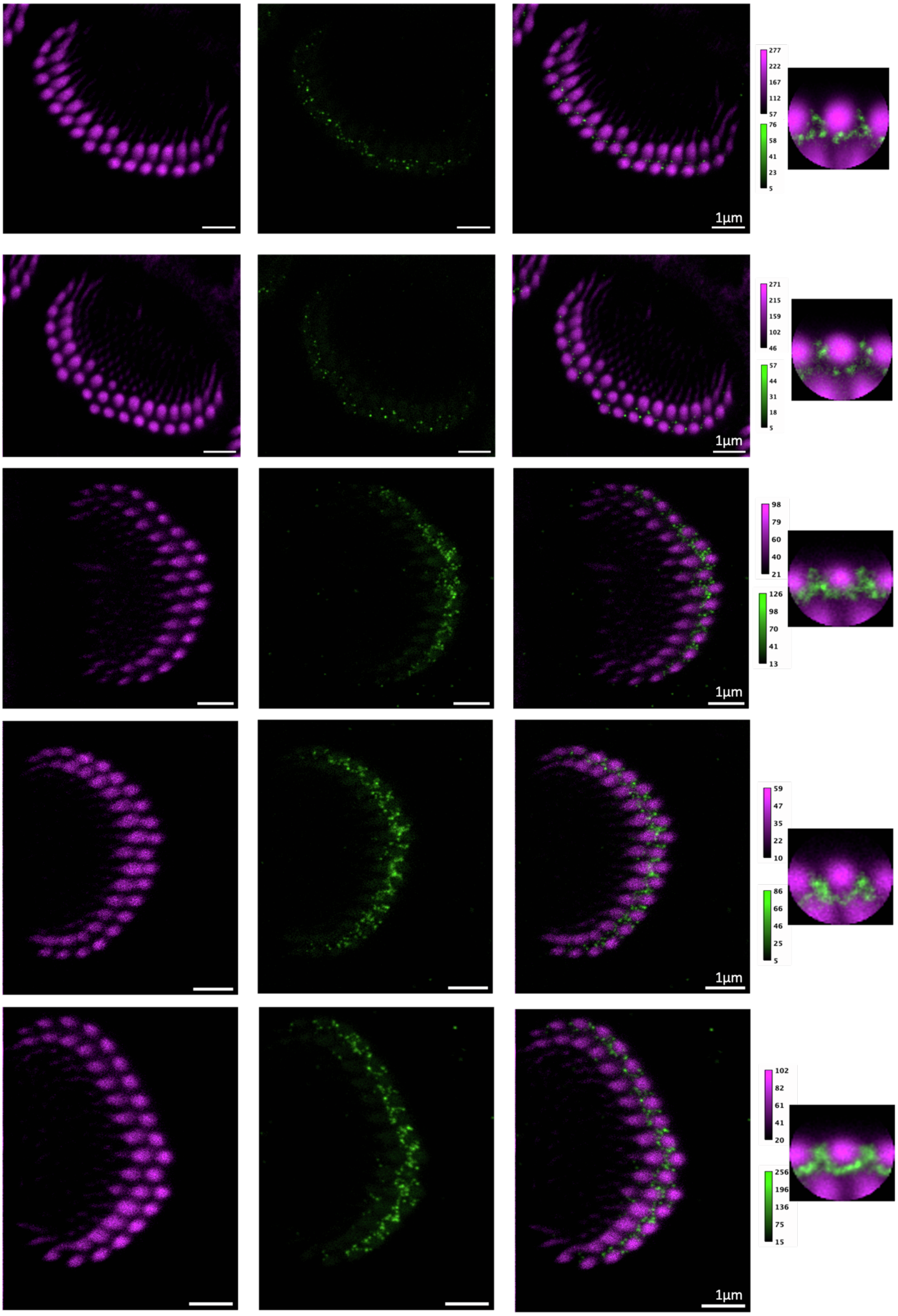
1µm
277
222
167
112
57
76
58
41
23
5
1µm
271
215
159
102
46
57
44
31
18
5
1µm
98
79
60
40
21
126
98
70
41
13
1µm
59
47
35
22
10
86
66
46
25
5
1µm
102
82
61
41
20
256
196
136
75
15

B OHCs ADGRV1 extra

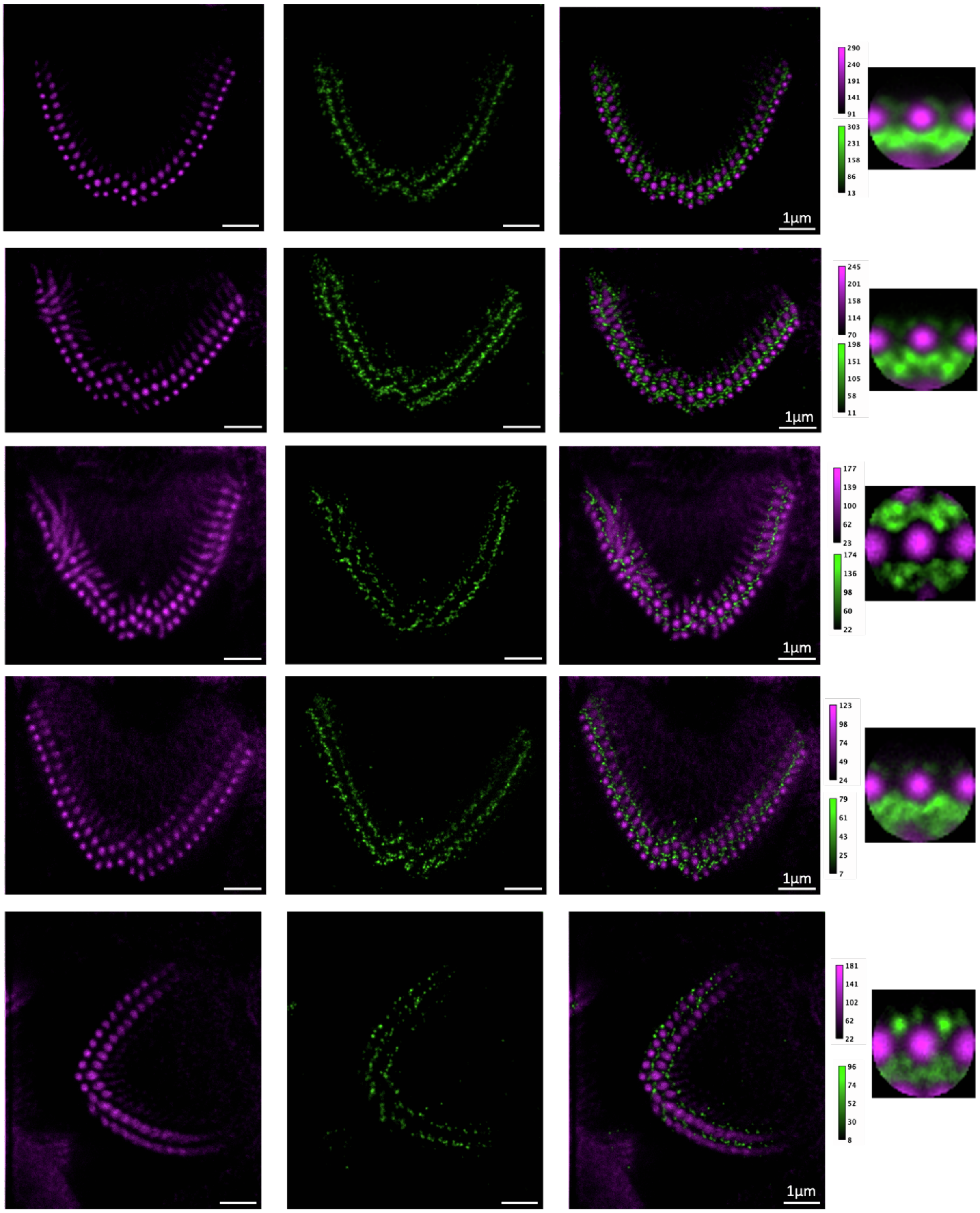

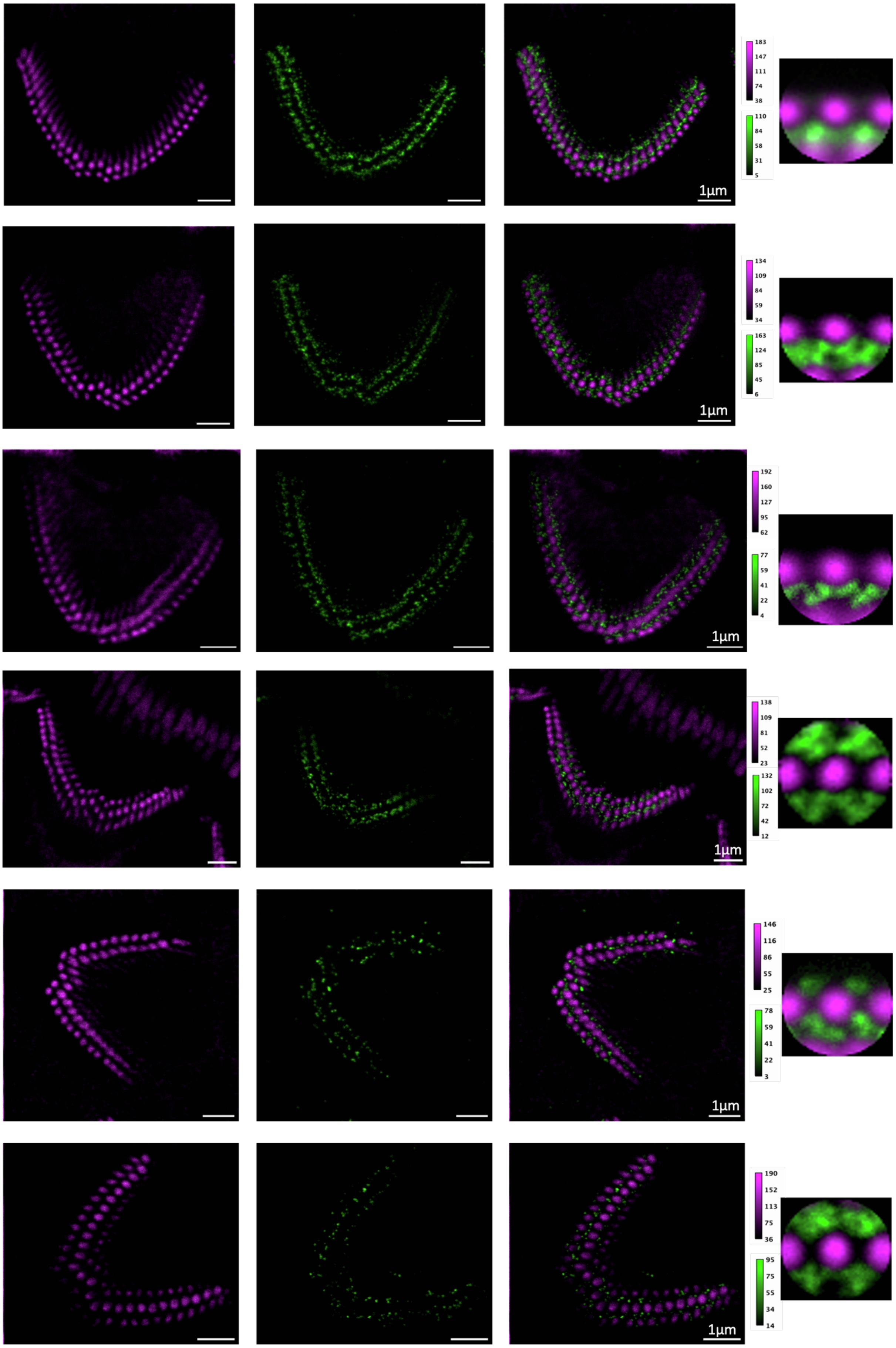

1µm
183
147
111
74
38
110
84
58
31
5
1µm
134
109
84
59
34
163
124
85
45
6
1µm
192
160
127
95
62
77
59
41
22
4
1µm
138
109
81
52
23
132
102
72
42
12
1µm
146
116
86
55
25
78
59
41
22
3
1µm
190
152
113
75
36
95
75
55
34
14

C IHCs ADGRV1 intra

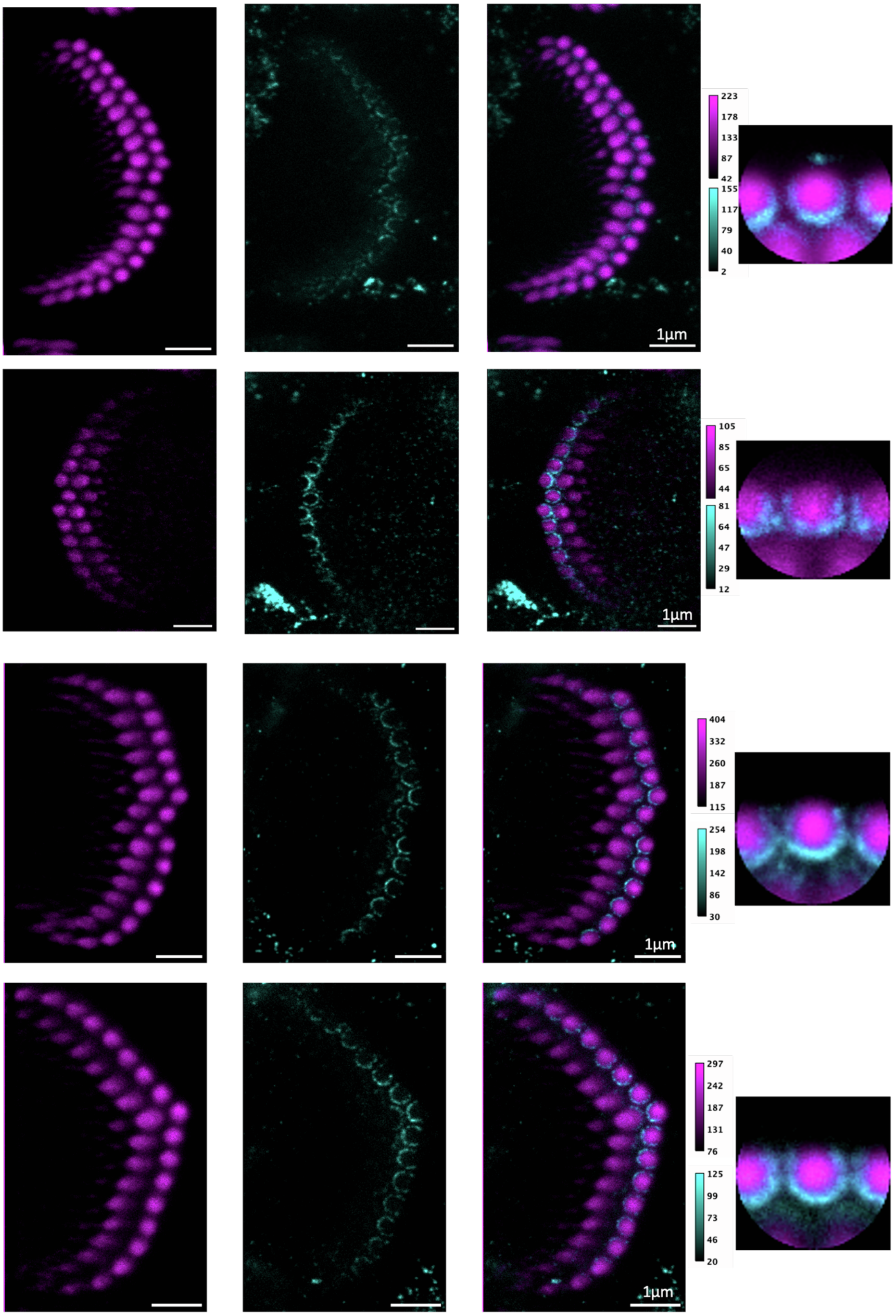

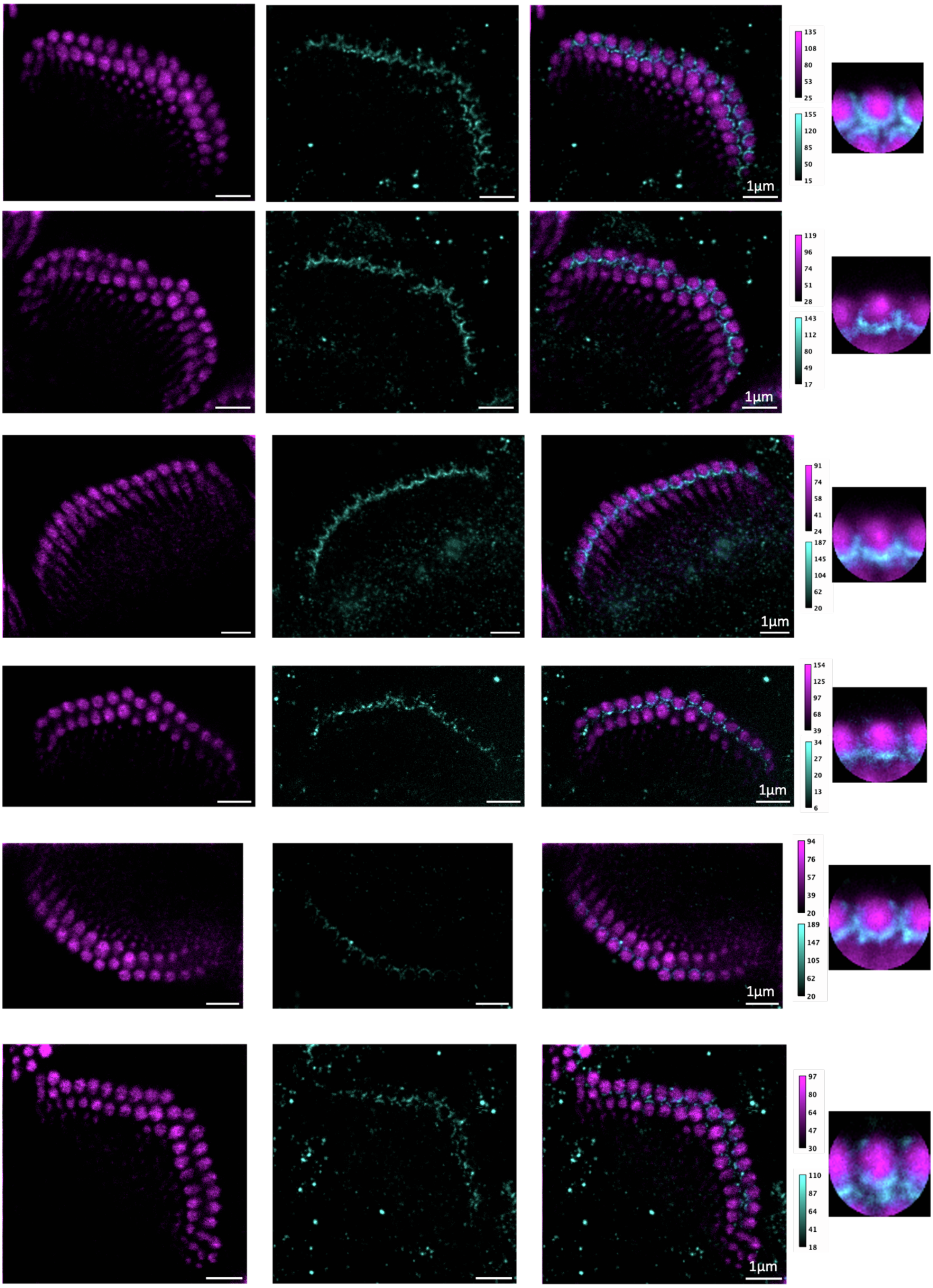
1µm
135
108
80
53
25
155
120
85
50
15
1µm
119
96
74
51
28
143
112
80
49
17
1µm
91
74
58
41
24
187
145
104
62
20
1µm
154
125
97
68
39
34
27
20
13
6
1µm
94
76
57
39
20
189
147
105
62
20
1µm
97
80
64
47
30
110
87
64
41
18

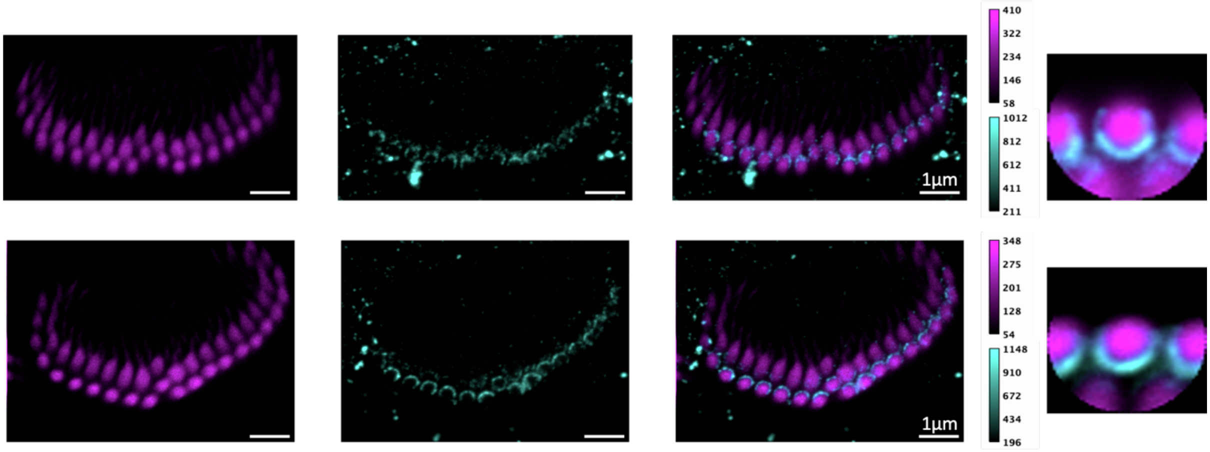

1µm
410
322
234
146
58
1012
812
612
411
211
1µm
348
275
201
128
54
1148
910
672
434
196

D OHCs ADGRV1 intra

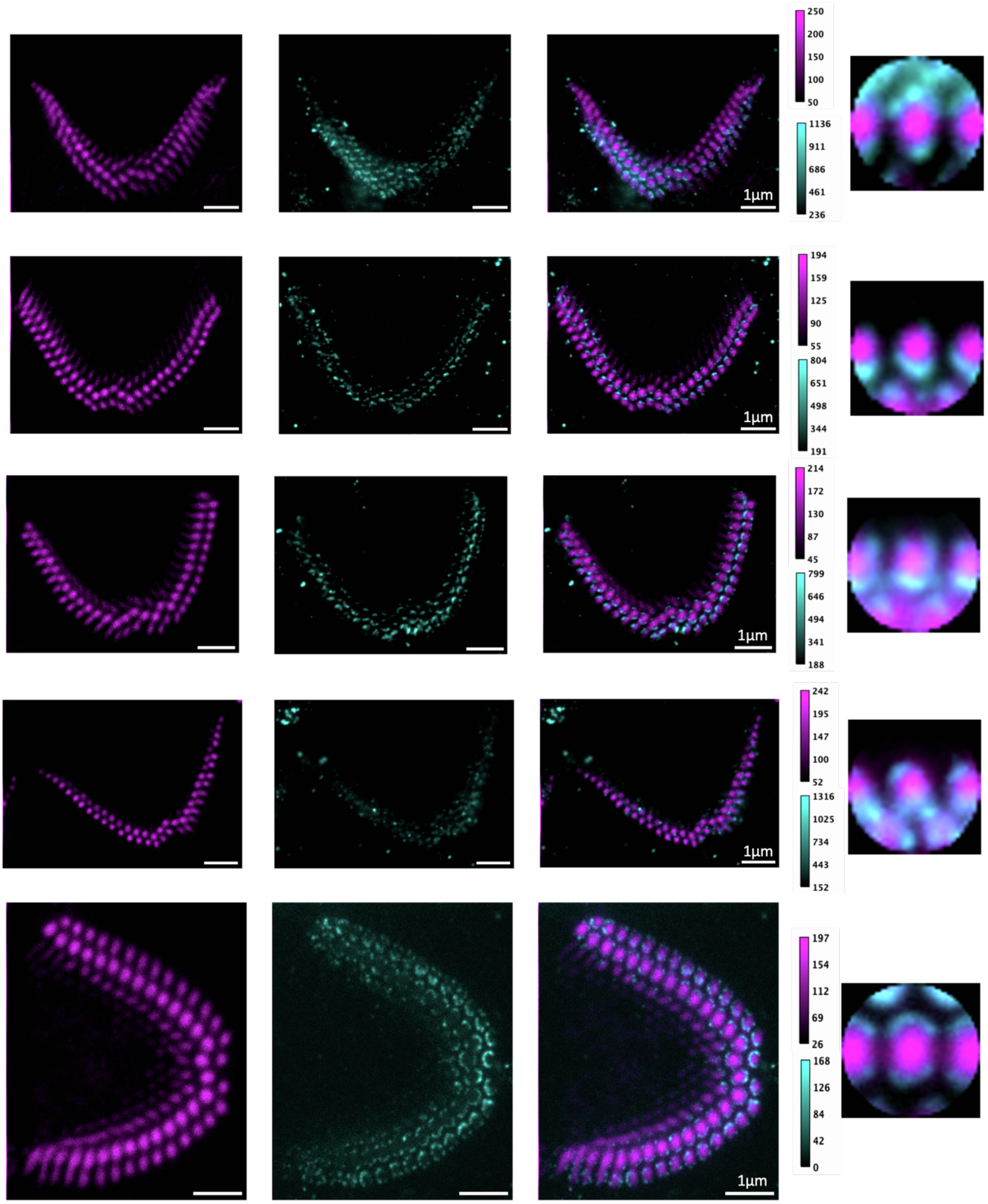

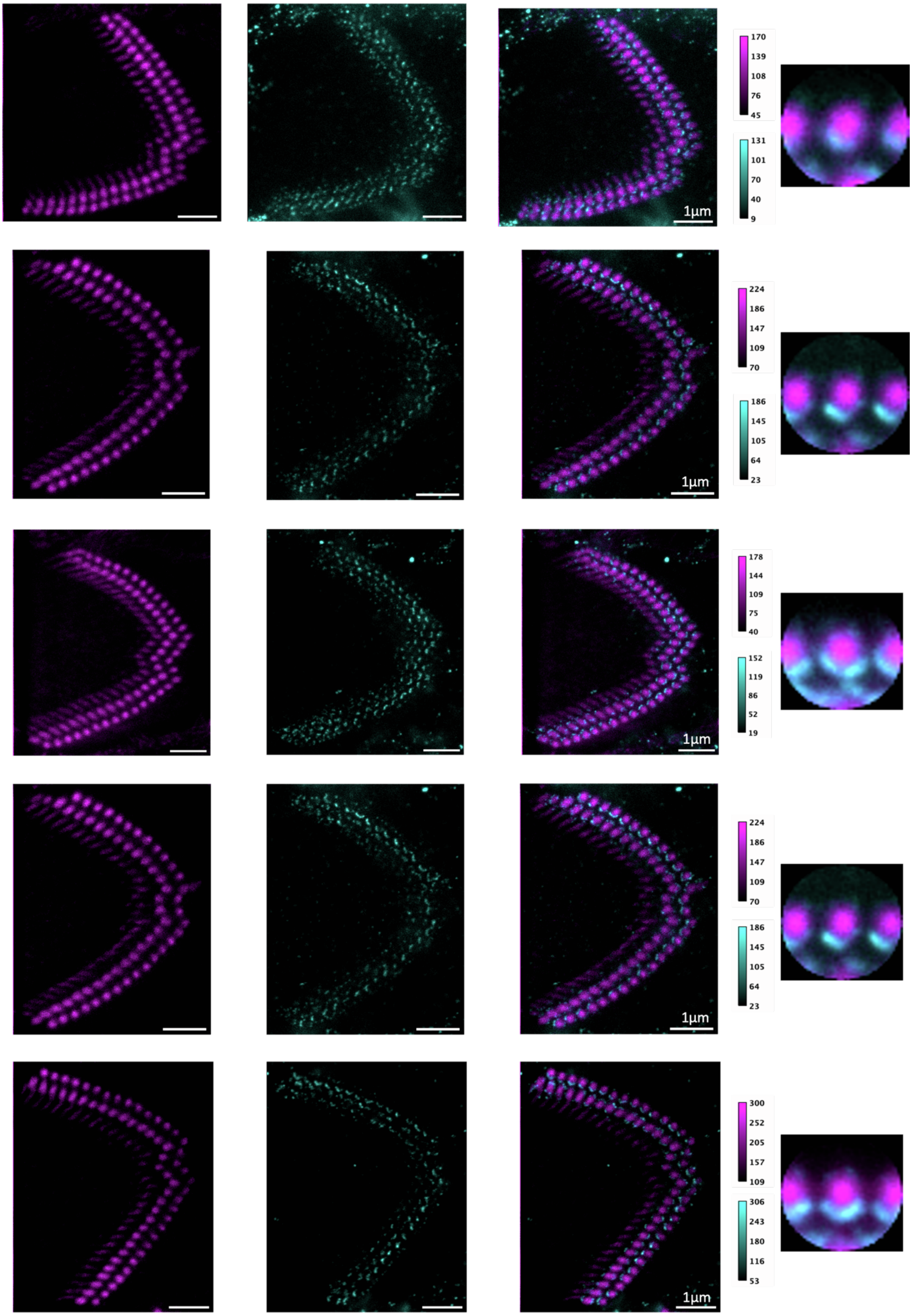
1µm
170
139
108
76
45
131
101
70
40
9
1µm
224
186
147
109
70
186
145
105
64
23
1µm
178
144
109
75
40
152
119
86
52
19
1µm
224
186
147
109
70
186
145
105
64
23
1µm
300
252
205
157
109
306
243
180
116
53

E IHCs PDZD7

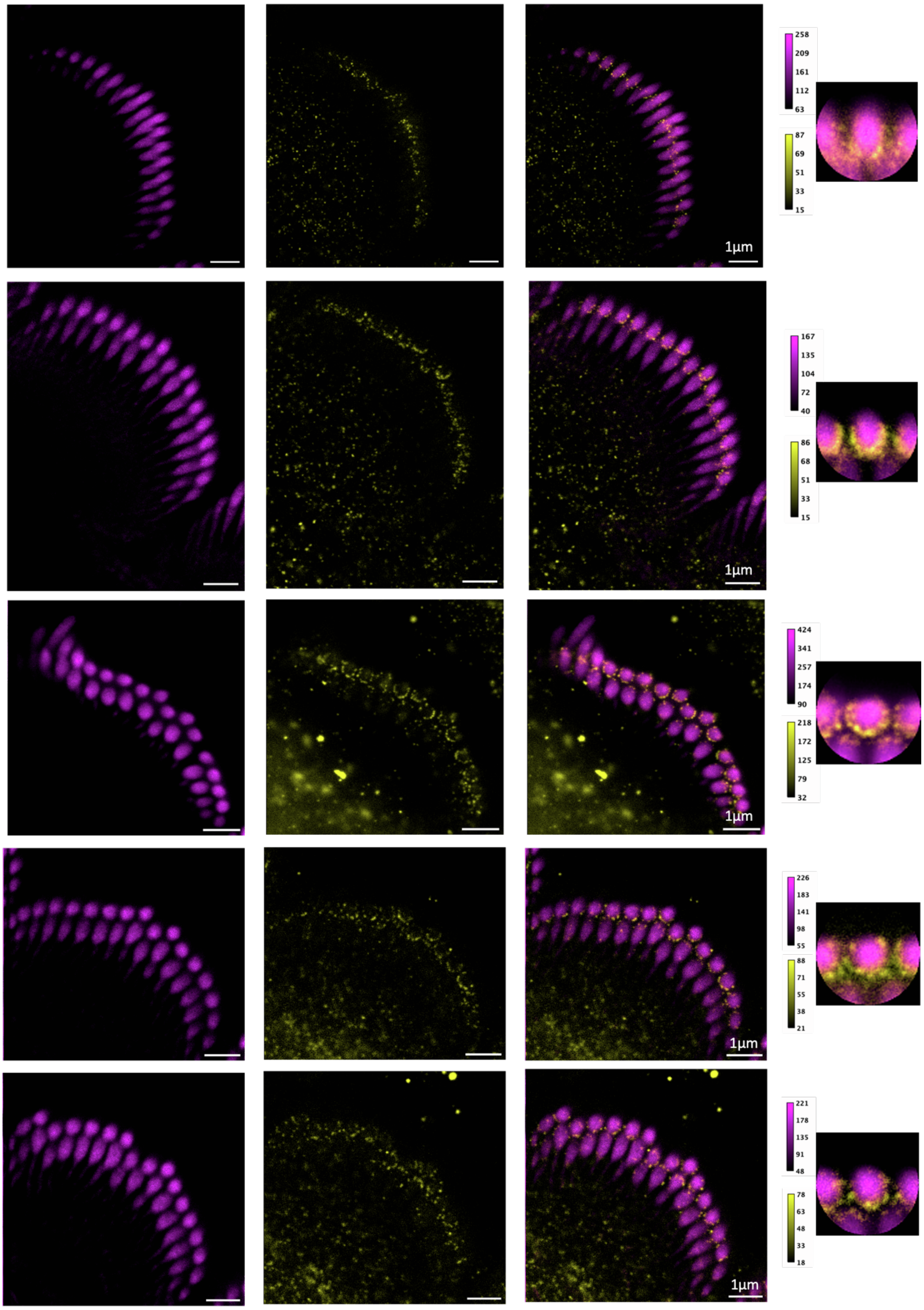
1µm
258
209
161
112
63
87
69
51
33
15
167
135
104
72
40
86
68
51
33
15
424
341
257
174
90
218
172
125
79
32
226
183
141
98
55
88
71
55
38
21
221
178
135
91
48
78
63
48
33
18

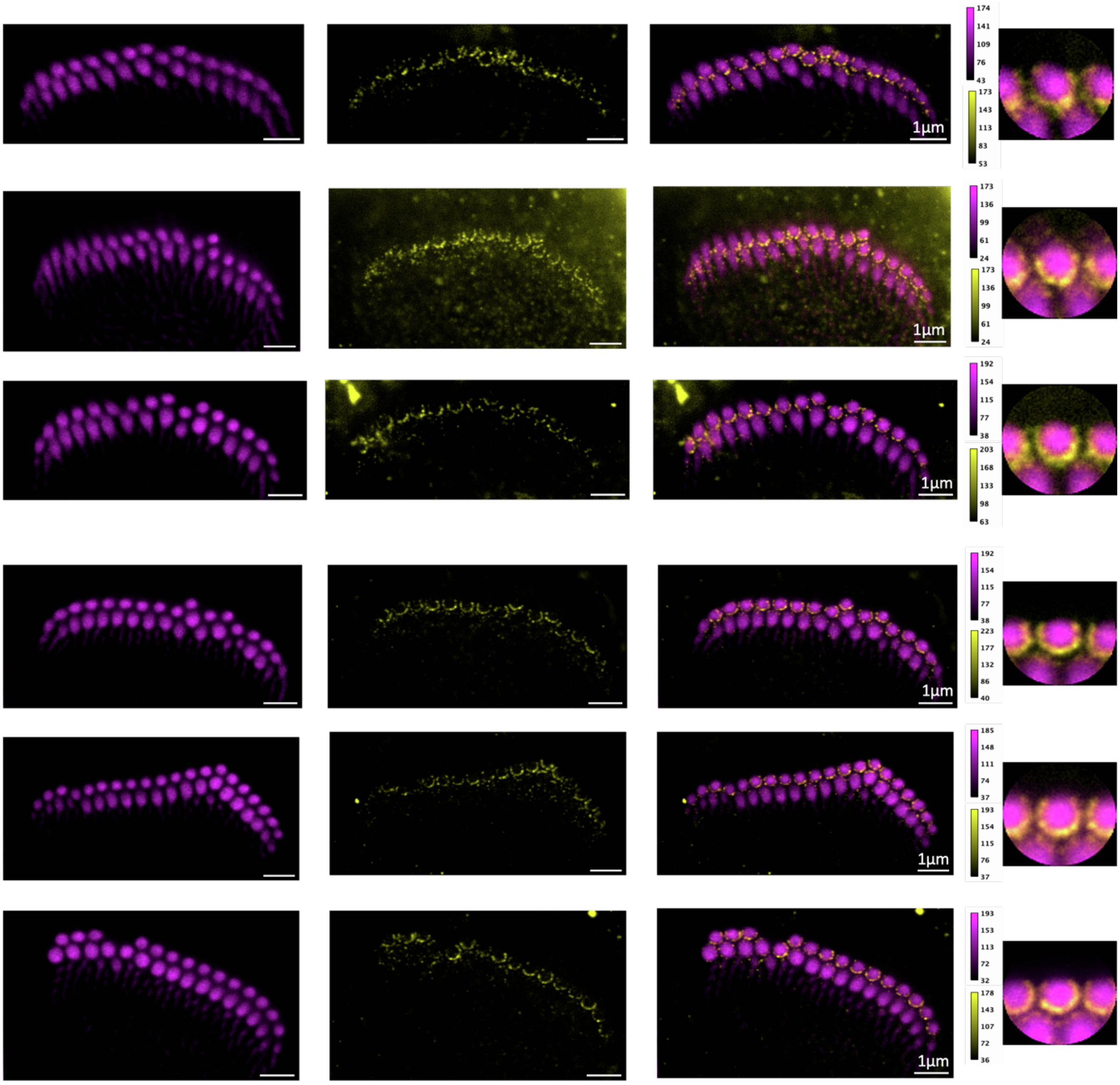
1μm
174 141 109 76 43
173 143 113 83 53
1μm
173 136 99 61 24
173 136 99 61 24
1μm
192 154 115 77 38
203 168 133 98 63
1μm
192 154 115 77 38
223 177 132 86 40
1μm
185 148 111 74 37
193 154 115 76 37
1μm
193 153 113 72 32
178 143 107 72 36

F OHCs PDZD7

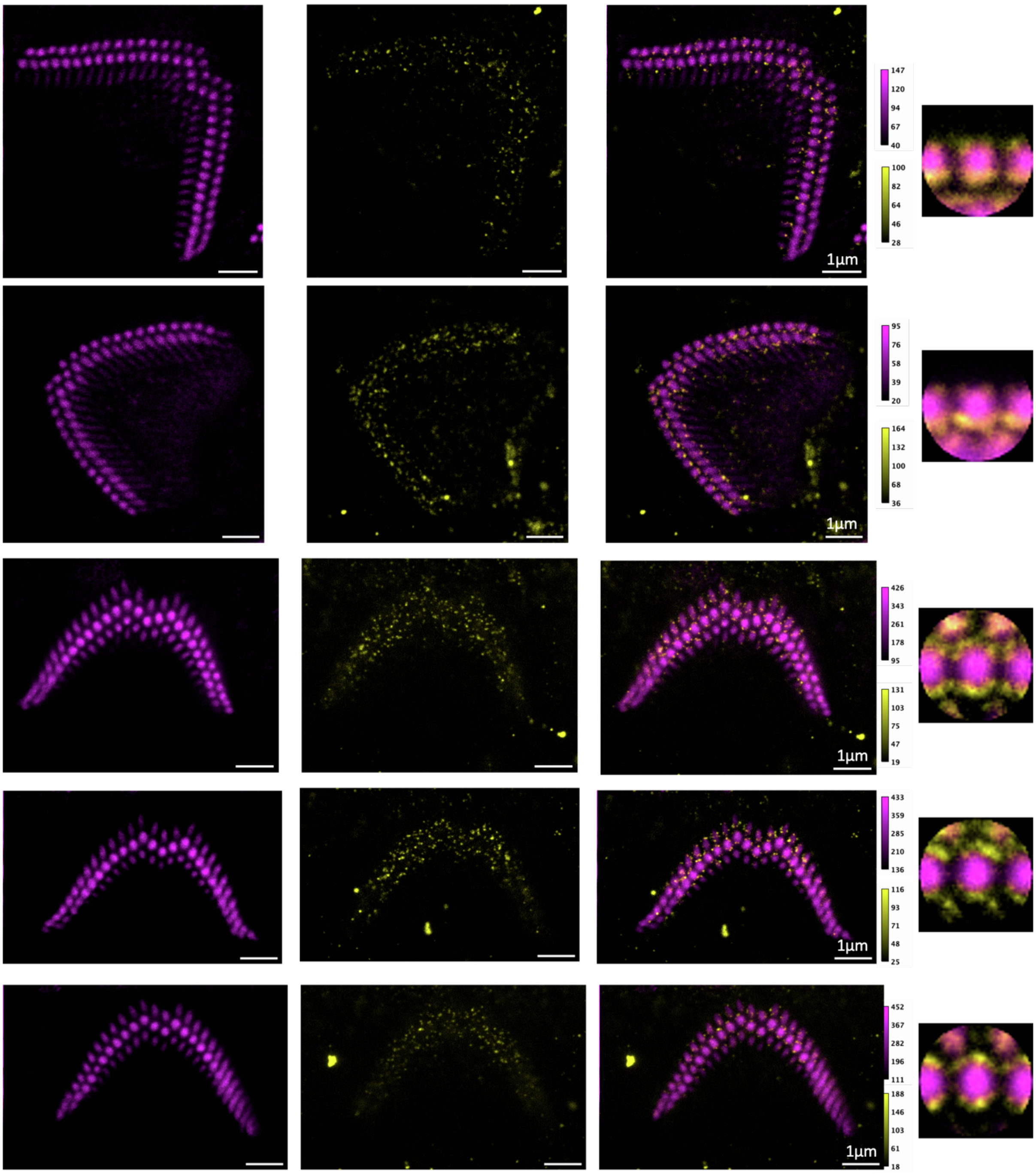
1µm
147
120
94
67
40
100
82
64
46
28
95
76
58
39
20
164
132
100
68
36
426
343
261
178
95
131
103
75
47
19
433
359
285
210
136
116
93
71
48
25
452
367
282
196
111
188
146
103
61
18

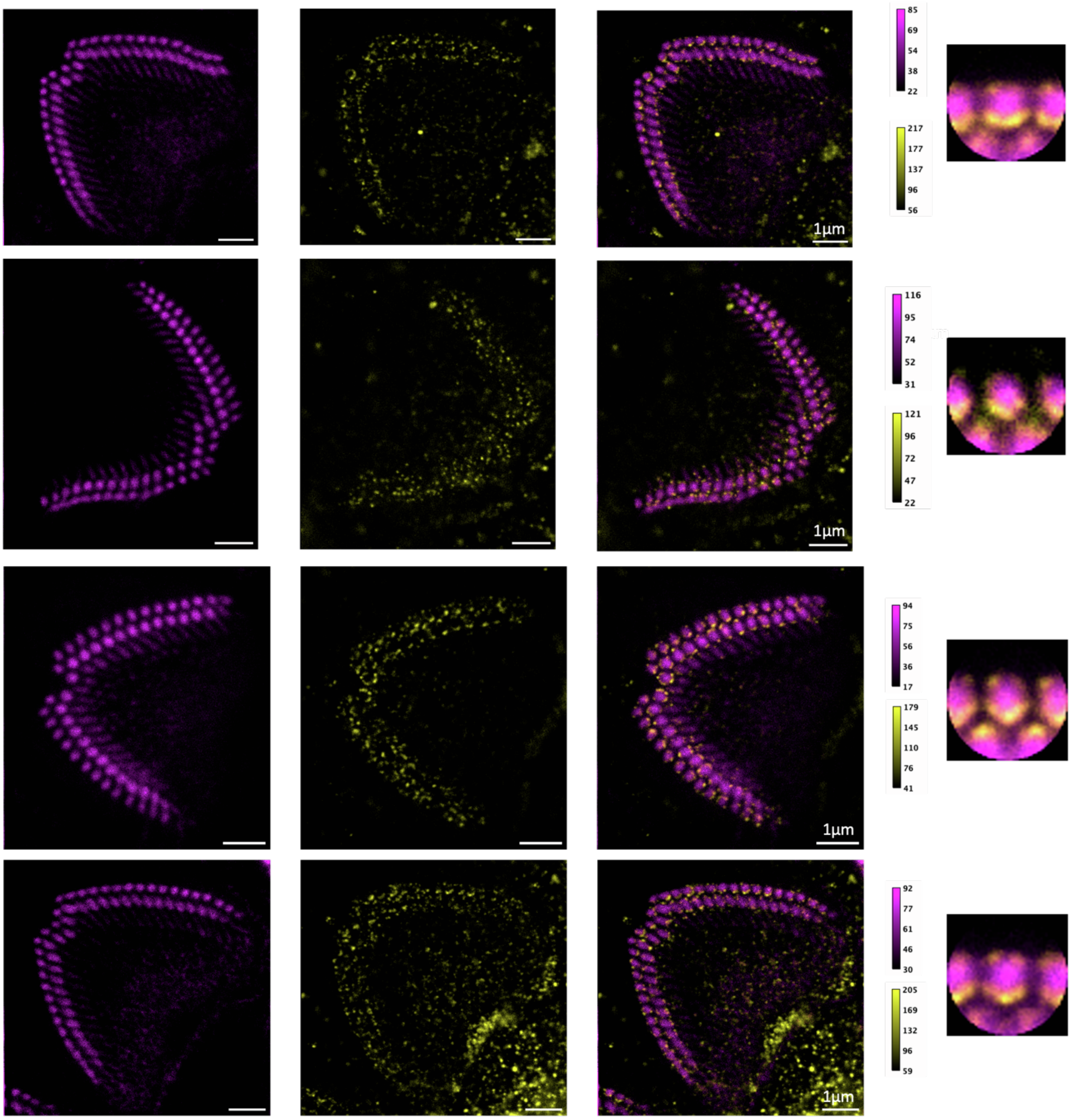

1µm
85
69
54
38
22
217
177
137
96
56
1µm
116
95
74
52
31
121
96
72
47
22
1µm
94
75
56
36
17
179
145
110
76
41
1µm
92
77
61
46
30
205
169
132
96
59

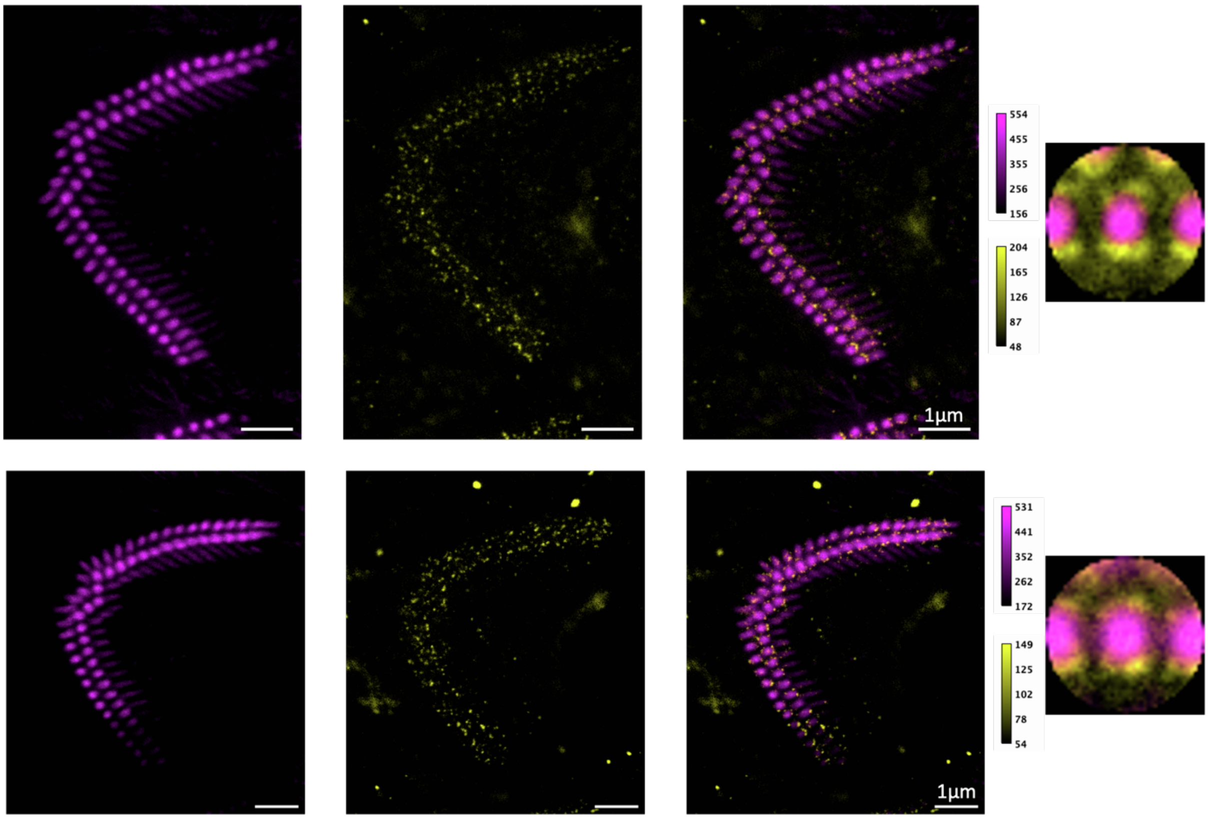
554
455
355
256
156
204
165
126
87
48
1µm
531
441
352
262
172
149
125
102
78
54
1µm

Figure S4. Additional individual IHCs and OHCs with immunolabeling of ADGRV1 extracellular and intracellular regions and PDZD7 in P5 mouse

Two-color STED microscopy imaging of ADGRV1 extracellular and intracellular regions, PDZD7 and actin in P5 mouse. Scale bar, 1µm. The intensity scales are reported for the two colors. Top views.

(A) IHCs STED images with labeling of actin (1st column, purple), of ADGRV1 extracellular region (2nd column, green), and composite image (3rd column). Sum projection of aligned individual stereocilia of the 1st or 2nd rows after normalization of their orientation the (4th column).

(B) OHCs STED images with labeling of actin (1st column, purple), of ADGRV1 extracellular region (2nd column, green), and composite image (3rd column). Sum projection of aligned individual stereocilia of the 1st or 2nd rows after normalization of their orientation the (4th column).

(C) IHCs STED images with labeling of actin (1st column, purple), of ADGRV1 intracellular region (2nd column, cyan), and composite image (3rd column). Sum projection of aligned individual stereocilia of the 1st or 2nd rows after normalization of their orientation the (4th column).

(D) OHCs STED images with labeling of actin (1st column, purple), of ADGRV1 intracellular region (2nd column, cyan), and composite image (3rd column). Sum projection of aligned individual stereocilia of the 1st or 2nd rows after normalization of their orientation the (4th column).

(E) IHCs STED images with labeling of actin (1st column, purple), of PDZD7 (2nd column, yellow), and composite image (3rd column). Sum projection of aligned individual stereocilia of the 1st or 2nd rows after normalization of their orientation the (4th column).

(F) OHCs STED images with labeling of actin (1st column, purple), of PDZD7 (2nd column, yellow), and composite image (3rd column). Sum projection of aligned individual stereocilia of the 1st or 2nd rows after normalization of their orientation the (4th column).
